# A Possibility-Space Derivation of a Planck-Softened Correlation Kernel for Holographic Entropy Deficit

**DOI:** 10.3390/e28101142

**Published:** 2026-10-17

**Authors:** Aaron Adair

**Affiliations:** Department of Physics, Massachusetts Institute of Technology, Cambridge, MA 02139, USA; adairaar@mit.edu

**Keywords:** maximum entropy, holographic principle, emergent spacetime, Lorentzian correlation kernel, Bekenstein–Hawking entropy, Klein–Gordon equation, Fisher information geometry, Einstein field equation, non-AdS holography, information theory

## Abstract

I derive a Lorentzian correlation kernel from a maximum-entropy principle on an abstract possibility space, assuming no spacetime or field theory. Maximizing Shannon entropy over positive phase rates at fixed mean gives $p\left(\omega \right)=\Omega^{-1}e^{-\omega /\Omega }$, and averaging relative phase coherence yields $\rho \left(\sigma \right)=1/\left(1+\Omega^{2}\sigma^{2}\right)$. Given a posited array of pairwise comparison intervals, classical multidimensional scaling returns a screen, its metric, and its dimension; the dimension is an output, and the test can fail, which makes the existence of a geometry falsifiable in principle. A postulated finite invariant speed supplies the Lorentzian cone. Under a stated locality assumption the Fisher coordinate of the ensemble is a shift-symmetric scalar that admits no potential within the Fisher sector; with Lovelock’s theorem this gives $G_{\mu \nu }+\Lambda g_{\mu \nu }=\left(8\pi G/c^{4}\right)T_{\mu \nu }$, with the coupling normalized thermodynamically and *G* an input. A Gaussian treatment of the patches solves for the correlation scale, giving $\lambda_{G}≃1.302\;\ell_{P}$. Treating Planck patches as binary variables on a square lattice instead sets $\lambda =\sqrt{2}\;\ell_{P}$, the largest scale binary patches can realize, before any entropy comparison. At that scale the maximum-entropy Ising multi-information agrees with 1−1/(4ln2)=0.6393, the Bekenstein–Hawking deficit, within 0.05%: transfer-matrix bounds give 0.6135, and 0.6389 under one assumption; a pre-registered extrapolation gives 0.6391. No anti-de Sitter asymptotics are used.

## 1. Introduction

The Bekenstein–Hawking entropy $S_{\mathrm{BH}}=k_{B}A/4\ell_{P}^{2}$ [1,2] assigns 1/(4ln2)≃0.3607 bits per Planck area to a horizon. One independent bit per Planck-area patch overcounts this by a factor 4ln2, so correlations among the patches must lower the entropy from one bit per patch to 1/(4ln2) bits. The shortfall,

(1) $$c_{\mathrm{total}}=1-\frac{1}{4\ln2}≃0.6393$$

bits per patch, is the shared information (multi-information) the patches must carry. This paper builds a minimal information-theoretic construction that produces such a deficit. It starts from an abstract possibility space carrying positive phase-rate variables, with no spacetime or field theory assumed at the outset, and arrives at the kernel

(2) $$\rho \left(d\right)=\frac{\lambda^{2}}{d^{2}+\lambda^{2}},$$

finite at zero separation and falling as $\lambda^{2}/d^{2}$. Its scale λ is either solved for from $c_{\mathrm{total}}$ (Gaussian patches) or fixed in advance at the largest value binary patches can realize (binary patches), as Section 7 and Section 8 describe.

### 1.1. What Is New in This Paper

The ingredients are standard: the exponential is the maximum-entropy density on positive quantities with fixed mean [3], its characteristic function is Lorentzian, and Lorentzian covariance kernels are common in signal processing and geostatistics [4]. The contributions lie in the combination.

(i) *A kernel from inference.* A stated maximum-entropy problem on an abstract set, with every input listed in Table 1, gives the Lorentzian kernel (Section 2, Section 3 and Section 4).
(ii) *A geometry that can fail.* Given a correlation matrix, classical multidimensional scaling returns a screen (the configuration of patches), its metric, and its dimension. The embeddability test fails on a wrapped lattice at every correlation length, and the wrapped 4×4 target stops being a correlation matrix above a computed threshold (Section 5.2); arrays with no low-dimensional structure fail outright (Section 5.1).
(iii) *The Einstein form from Fisher geometry.* Under a locality assumption the Fisher coordinate of the phase-rate family is a shift-symmetric scalar that admits no potential within the Fisher sector, and Lovelock’s theorem [5] fixes the Einstein form (Section 6). The form uses no Rindler horizon, Unruh temperature, or area law; the coupling constant is fixed by the Clausius relation [6].
(iv) *A scale fixed in advance, and a close agreement.* On a square lattice of binary patches with spacing *a*, binary realizability caps the correlation length at $\lambda =\sqrt{2}\;a$ exactly; the scale is set at that edge, $\lambda_{\mathrm{bin}}=\sqrt{2}\;a$, before any entropy comparison. At that scale the maximum-entropy Ising multi-information agrees with $c_{\mathrm{total}}=0.6393$ to 0.05%: transfer-matrix bounds reach 0.6135 unconditionally and 0.6389 under one stated assumption about the thermodynamic limit, and a pre-registered extrapolation gives 0.6391. Whether the agreement is exact is open (Section 8.6). A Gaussian treatment instead solves for its scale, $\lambda_{G}≃1.302\;a$, which is a calibration (Section 7.1).
(v) *A time orientation without a background time.* The reconstructed screens can be ordered by their accessible holographic information; the ordering has a first element at the metric-onset scale, and it orients the time coordinate without supplying its metric (Section 10).
(vi) *A non-AdS setting.* No anti-de Sitter bulk or boundary conformal field theory is assumed, so FLRW, de Sitter, and flat settings are in scope.

### 1.2. What Is Assumed, and What Follows from It

Table 1 lists the inputs adopted without derivation, with their type: a *postulate*, an *identification* of units or scales, an *empirical input*, a *modeling choice*, or a *falsifiable claim*. Table 2 lists what follows and in what sense. Two empirical inputs enter: Newton’s constant, through $a=\ell_{P}$ and the coupling, and the Bekenstein–Hawking coefficient. One value is fitted to the latter, $\lambda_{G}$; the pairwise scale of Section 7 is solved for in the same way and serves only as a comparison. No other entry is adjusted to agree with a measured quantity. The separation array and the finiteness of the invariant speed are posited, one-point dynamics assumes locality, Λ is undetermined, the dimension is measured, the phase-rate ensemble is classical, and whether $c_{\infty }$ equals $c_{\mathrm{total}}$ exactly is open. The tables can be read after Section 5; symbols are collected in Table A1.

### 1.3. Relation to AdS and Non-AdS Holography

The holographic principle [7,8,9] has its most precise realization in the AdS/CFT correspondence [10], where a gravitational theory in asymptotically anti-de Sitter space is dual to a conformal field theory on its boundary. Two features of that setting limit its reach. The duality needs a timelike conformal boundary, which the observed universe lacks: local Minkowski limits, FLRW cosmology, quasi-de Sitter inflation, and late-time de Sitter-like horizons are none of them asymptotically AdS. Attempts to carry the construction to de Sitter space place the dual on a spacelike boundary at future infinity, where the putative field theory is Euclidean and need not be unitary [11]; the status of de Sitter holography remains open.

The construction here uses holography more modestly. It takes one input from it, the Bekenstein–Hawking area law, and uses it as a calibration: the correlation deficit a screen must carry. No boundary dual, no asymptotic region, and no conformal symmetry are assumed. The screen itself is reconstructed from correlations (Section 5), so the same construction applies to a horizon cross-section, a cosmological screen, or a flat region. The entropy line of the argument runs

(3) $$\begin{array}{cc} \mathrm{maximum}\text{-}\mathrm{entropy}\;\mathrm{phase}\;\mathrm{rates} & \to \mathrm{finite}\;\mathrm{inverse}\text{-}\mathrm{square}\;\mathrm{correlation}\;\mathrm{kernel}\\ & \to \mathrm{distance}\to \mathrm{area}\;\mathrm{entropy}\to \mathrm{entropic}\;\mathrm{time}\;\mathrm{orientation}, \end{array}$$

and the dynamics line branches from the distance step to the Einstein form (Section 6). What this gives up is the dynamical content of a dual theory: nothing here plays the role of the boundary CFT, and the entropy is matched, not computed from a microscopic Hamiltonian.

### 1.4. Relation to Space-from-Entanglement Programs

Several programs derive geometry from quantum information or from a discrete pre-geometric structure. Each meets a characteristic difficulty, and the construction here is designed around those difficulties.

Entanglement and bulk connectivity.

Ryu and Takayanagi relate bulk minimal areas to boundary entanglement [12], and Van Raamsdonk argues that bulk connectivity is built from that entanglement [13]; see [14] for a survey of open problems. Both presuppose a holographic dual, so they explain how a known bulk is encoded, not how a geometry arises where no dual is given. Tensor-network models [15,16] make the encoding explicit, but the network graph, and with it the bulk geometry, is chosen at the outset. Here the analog of the graph is an output: the correlation matrix is tested for Euclidean embeddability, the dimension is read off as rankB, and the test can fail (Section 5.1 and Section 5.2).

Geometry from mutual information.

Cao, Carroll, and Michalakis reconstruct space from the entanglement of a state in Hilbert space by multidimensional scaling [17], the same reconstruction step used in Section 5. Three issues remain open in that program: the map from mutual information to distance is chosen, not derived; the tensor factorization of Hilbert space that defines the subsystems is assumed, and Hilbert spaces of equal dimension are isomorphic, so the choice carries the geometry; and perturbations of the state give a *spatial* analog of the Einstein equation, with spacetime covariance assumed. Here the kernel, and hence the correlation-to-distance map $d=\lambda \sqrt{\rho^{-1}-1}$, follows from maximum entropy (Section 4); the elements of P are abstract possibilities with no tensor-product structure, so the factorization question is deferred to a quantum treatment; and the form of the full spacetime equation follows from Fisher geometry and Lovelock’s theorem (Section 6), under the assumptions of Table 1.

Thermodynamic derivations.

Jacobson derives the Einstein equation from the Clausius relation on local Rindler horizons [6,18]. The derivation presupposes a spacetime, local horizons, the Unruh temperature, and an entropy proportional to area, and the last of these is the quantity an emergent account would want to explain. Here the *form* of the field equation is obtained without horizons, temperatures, or an area law, and the Clausius relation is used only for the constant κ (Section 6).

Entropic force.

Verlinde treats gravity as an entropic force on a test body at a holographic screen [19]. A genuinely thermal force would decohere superposed masses, and neutron and matter-wave interferometry constrain that sharply [20,21,22]. The screens are also postulated. Here nothing acts on a test body: $\rho_{ij}$ is a statistical expectation over the phase-rate distribution, the screen is reconstructed, and gravity enters through a field equation. The decoherence objection therefore has no direct target, although whether the underlying degrees of freedom stay coherent is open (Section 11).

Causal sets.

Causal set theory [23,24] is the closest structural analog: both are discrete at the Planck scale and recover Lorentzian geometry from pre-geometric data. The primitive relation differs—causal order there and correlation here—and so does the inverse problem. Most partial orders are far from manifold-like, since three-layer orders dominate the count [25], so manifold-likeness has to come from dynamics, and a complete dynamics is still sought; recovering dimension and action uses counting estimators (Myrheim–Meyer, Benincasa–Dowker). Here the inverse problem is classical scaling with an exact criterion, B⪰0, and the dimension is an eigenvalue count. The cost is the converse: causal order is not primitive, and the Lorentzian cone comes from the invariant-speed postulate.

Information geometry.

Caticha’s entropic dynamics applies maximum entropy to abstract parameter spaces and obtains quantum mechanics as inference [26,27,28]. The Fisher geometry of Section 6 belongs to the same family, used here for geometry and gravity. Neukart, Marx, and Vinokur couple local entropy production to curvature through a non-minimally coupled scalar and recover $S_{\mathrm{BH}}$ in stationary limits [29]. Here the scalar φ=logΩ is the affine Fisher coordinate of the phase-rate family, and its shift symmetry φ→φ+C excludes a coupling $\xi R\varphi^{2}$ (Section 6).

### 1.5. Summary of the Comparison

Table 3 collects the comparison. What the approach offers in exchange for the costs listed in Section 1.2 is a single chain—from one inference problem to a kernel, a reconstructed screen, and the form of the Einstein equation—in which each step can be checked and several can fail.

**Figure 1:**
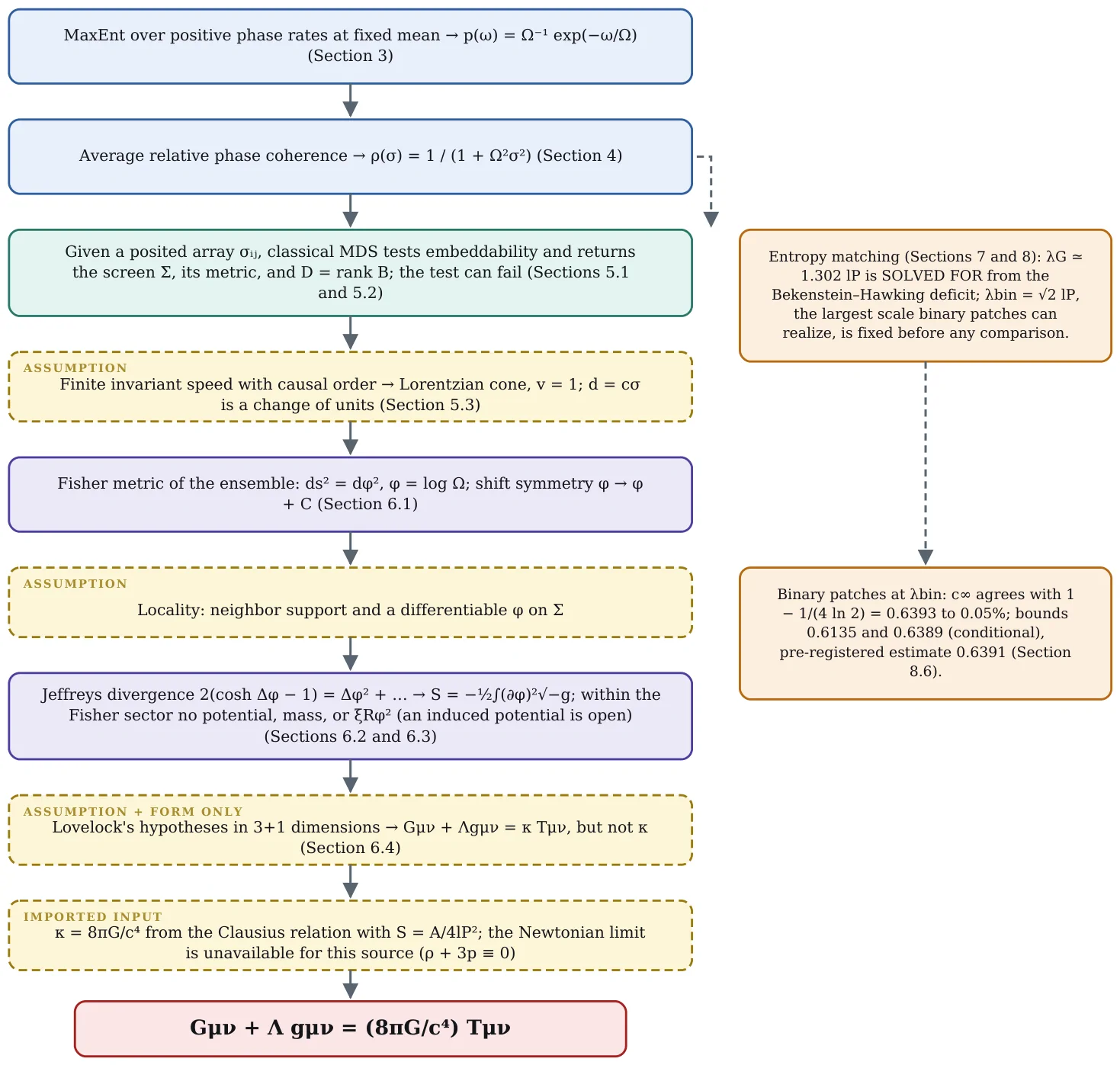
Derivation chain from maximum entropy on possibility space P to the Einstein field equation. Blue: information-theoretic core (Section 3 and Section 4). Teal: reconstruction, signature, and length unit (Section 5). Indigo: Fisher geometry and Lovelock uniqueness (Section 6). Orange: entropy matching (Section 7 and Section 8). Dashed amber boxes: the invariant-speed postulate, the locality assumption, Lovelock’s hypotheses, and the coupling normalization, four of the inputs of Table 1. Dashed arrows: the side branch to the entropy matching. Red: the field equation.

Section 2, Section 3 and Section 4 derive the phase-rate distribution and the kernel. Section 5 reconstructs the screen and fixes its signature and length unit. Section 6 places a field on the reconstructed screen and obtains the form of the Einstein equation. Section 7 and Section 8 carry out the entropy matching, Gaussian and binary. Section 9 treats the domain of the metric description, Section 10 the time orientation, and Section 11 the limitations. Figure 1 maps the chain.

## 2. Possibility Space and Phase-Rate Variables

Let P be an abstract possibility space with elements i,j,k,…. It is not assumed to be space, spacetime, a manifold, or an embedded lattice: it is a domain of distinguishable possibilities, and the framework specifies a probability calculus over them without asking what they physically are.

Patches, not points.

The elements of P are *patches*, subsets of positive measure, for two reasons. A probability measure gives positive weight to at most countably many points, so on an uncountable space almost every point has probability zero and cannot be a distinguishable possibility that carries weight. And an observer with a finite information capacity of *k* bits resolves at most $2^{k}$ alternatives, so any state of knowledge is a set of positive measure and positive entropy. A disjoint family of positive-measure sets is in turn at most countable, since at most *n* of them can have measure above 1/n [30]. So a possibility space may be uncountable, but its partition into distinguishable patches is not: the correlation structure $\rho_{ij}$ is a countable array, the entropies used below are defined on a countably generated σ-algebra, and the lattice sums of Section 7 and Section 8 are well defined.

Phases and their comparison.

To each patch associate a phase that advances with a parameter σ at rate $\omega_{i}\geq 0$,

(4) $$z_{i}\left(\sigma \right)=e^{-i\omega_{i}\sigma }.$$

Here σ is the comparison parameter and $\omega_{i}$ an abstract frequency; neither is yet physical. Two patches are compared through their relative phase $z_{i}z_{j}^{*}=e^{-i(\omega_{i}-\omega_{j})\sigma }$. The complex phase is motivated by the path-integral weight $e^{iS/\hbar }$; under $\sigma \to -i\sigma_{E}$ it becomes the Euclidean weight $e^{-\omega_{i}\sigma_{E}}$.

Why the comparison oscillates.

Write the comparison as a function of the rate difference, z(Δω). Require that it be bounded and continuous, and that comparing *i* with *k* and *k* with *j* multiply: z(a+b)=z(a)z(b). The bounded continuous solutions are the characters of (R,+), $z\left(\Delta \omega \right)=e^{-i\Delta \omega \;\sigma }$ for real σ, which is the form above. Multiplicative composition is the modeling choice of Table 1; given it, the oscillating form is forced.

The inputs used so far are positive phase rates with a finite mean and multiplicative composition (Table 1, rows 1–3). Since the physical content of P will be exhausted by the correlations $\rho_{ij}$, configurations related by a relabeling of patches are the same state: Leibniz equivalence holds by construction, and the Hole Argument [31,32] has no purchase.

## 3. Maximum-Entropy Derivation of the Phase-Rate Distribution

Maximizing the Shannon entropy [33] $-\int_{0}^{\infty }p\ln p\;d\omega$ over densities on ω≥0 with fixed mean 〈ω〉=Ω gives [3]

(5) $$\begin{array}{c} p\left(\omega \right)=\frac{1}{\Omega }e^{-\omega /\Omega } \end{array}.$$

It is the least biased density over positive rates with a fixed mean. No thermal interpretation or spacetime action is assumed.

### Why the Constraint Is the Mean Rate

Shore and Johnson’s theorem [34] (see [35] on its scope) fixes the inference rule, not the constraint. The choice matters: 〈ω〉 gives the Lorentzian kernel, $\langle \omega^{2}\rangle$ a Gaussian-enveloped one. Three steps select the mean.

(i) Some constraint is required, since differential entropy on [0,∞) is unbounded. 〈logω〉 cannot come first, because logω needs a reference rate, which is the scale the first constraint must supply.
(ii) For independent subsystems the phases add, so the rates add, $\omega =\omega_{X}+\omega_{Y}$. A constraint that holds for the parts and for the whole must then be additive, and among the moments $E[\omega^{s}]$ only s=1 is: for independent exponentials $E\left[{\left(X+Y\right)}^{s}\right]/\left(E\left[X^{s}\right]+E\left[Y^{s}\right]\right)=\left(1+s\right)/2$.
(iii) Among additive quantities (the cumulants) the mean is the weakest: by Lyapunov’s inequality a finite variance implies a finite mean, and a Pareto ensemble with index 3/2 has a mean and no variance.

The load-bearing premise is additivity, listed in Table 1.

## 4. Correlation Kernel in Possibility Space

This section turns the density of Section 3 into a two-point correlation, using the comparison of Section 2 and the coherence definition of Table 1. For two patches i,j let $\sigma_{ij}\geq 0$ be their comparison interval, an abstract parameter that is not yet a distance. Define the correlation of two independently drawn rates as the averaged relative phase,

(6) $$\rho \left(\sigma \right)=E\left[\cos\left((\omega_{i}-\omega_{j})\sigma \right)\right]={\left|\psi \left(\sigma \right)\right|}^{2},\;\psi \left(\sigma \right)=\int_{0}^{\infty }p\left(\omega \right)e^{i\omega \sigma }\;d\omega =\frac{1}{1-i\Omega \sigma },$$

so that, with $\lambda_{\sigma }=1/\Omega$,

(7) $$\begin{array}{c} \rho \left(\sigma \right)=\frac{1}{1+\Omega^{2}\sigma^{2}}=\frac{\lambda_{\sigma }^{2}}{\sigma^{2}+\lambda_{\sigma }^{2}} \end{array}.$$

The kernel equals 1 at σ=0 and falls as $\lambda_{\sigma }^{2}/\sigma^{2}$: a nonsingular inverse-square tail. Given the comparison function, the exponential density, and this coherence definition, no other kernel results. By Bochner’s theorem [4,36] a stationary kernel is positive definite exactly when it is the Fourier transform of a non-negative measure,

(8) $$f\left(\sigma \right)=\int_{-\infty }^{\infty }e^{i\omega \sigma }\;d\mu \left(\omega \right),$$

as ρ is. Linear kernels 1−α|σ| fail this, and power laws ${\left|\sigma \right|}^{-\alpha }$ violate f(0)=1. The comparison parameter σ is measured in units of 1/Ω. Since σ enters only through Ωσ, the value of Ω is a choice of unit with no physical consequence; the content lies in the dimensionless ratio x=λ/a=c/(Ωa) of the correlation length to the patch spacing, fixed in Section 7.1 and Section 8.

Variational structure.

Treat σ formally as an evolution parameter. The kernel then obeys $\rho^{\prime \prime }=2\Omega^{2}\rho^{2}\left(3-4\rho \right)$, the equation of motion of a particle in the potential $V\left(\rho \right)=2\Omega^{2}\rho^{3}\left(\rho -1\right)$, and the kernel is the trajectory of zero total energy (Appendix C.1). Since *V* is reconstructed from the kernel, the orbit property is an identity; its content is *V*, which fixes how small departures from the kernel behave. Appendix C.3 promotes ρ(σ) to a field on the plane of pair separation and time, with a nonlinear Klein–Gordon equation and a fluctuation spectrum. The argument below does not depend on it.

Noise and the quantum of action.

The pair statistics of the ensemble also fix a noise floor, an exponential noise spectrum, and a minimum action per resolvable interval. No single value of the phase offset $\theta_{0}$ is used: the offset is unknown and, under the maximum-entropy ensemble, uniform on [0,2π), so the per-offset Fisher information $g(\theta_{0})$ is averaged uniformly over it (Equations (A16) and (A17)). At the one scale the kernel distinguishes, Ωσ=1, this gives 〈g〉=0.566 and the pure number $\hbar_{q}=\sqrt{\langle g\rangle }=0.752$, a value at a chosen scale (Table 2); the minimum energy of a mode is $\hbar_{q}\;\omega$, linear in its frequency (Appendix F). Nothing downstream uses $\hbar_{q}$; the linearity is what licenses E=ℏΩ in Section 6.

## 5. Reconstruction of the Screen from a Separation Array

This section takes as given a symmetric array of pairwise comparison intervals $\sigma_{ij}$, needed to turn the kernel ρ(σ) into a matrix $\rho_{ij}$, and, for the signature only, the finite-invariant-speed postulate (Table 1). Either the correlations $\rho_{ij}$ or the intervals $\sigma_{ij}$ may be taken as the data, since the kernel converts one into the other pair by pair. Nothing assumes that the $\sigma_{ij}$ are distances in any space. The theorem below tests whether they are, and if so in how many dimensions; that test is the content of the section, and the array can fail it. An assumed Euclidean screen is thereby replaced by an assumed array together with a test. Until Section 5.3, σ and $\lambda_{\sigma }=\Omega^{-1}$ are measured in units of 1/Ω, and no velocity enters.

### 5.1. The Reconstruction Theorem

The kernel is strictly monotone, so it inverts pair by pair,

(9) $$\sigma_{ij}=\Omega^{-1}\sqrt{\rho_{ij}^{-1}-1}.$$

**Theorem** **1** (Schoenberg [37])**.** *Let $Z_{ij}=\sigma_{ij}^{2}$, $P_{c}=I-N^{-1}11^{\;⊤}$, and $B=-\frac{1}{2}P_{c}ZP_{c}$. A configuration $\left\{u_{i}\right\}$ with $\sigma_{ij}=∥u_{i}-u_{j}∥$ exists if and only if B⪰0. It then needs rankB dimensions, is unique up to a rigid motion with absolute scale fixed by $\rho_{ij}$ and *Ω*, and is given by the rows of $VM^{1/2}$, where $B=VMV^{\;⊤}$ over the positive eigenvalues.*

The screen is the point set, its dimension an eigenvalue count, and its metric is fixed by the correlations. On random configurations in two and three dimensions the implementation returns the dimension and the separations to $2\times 10^{-15}$. On correlations generated by the model itself, (9) returns the posited $\sigma_{ij}$ exactly, so the test then examines the array itself; it has content independent of the construction when $\rho_{ij}$ is supplied by data (Section 5.4). rankB is the *embedding* dimension: patches on a sphere compared by chordal separation give 3, so rankB=2 for a horizon screen means the flat-patch approximation.

The dimension is read off.

An objection: rankB is a property of the array alone, so a reconstruction run on points placed in $R^{D}$ could simply return its input. Applied to arrays not built from coordinates, the procedure returns no Euclidean realization or only the trivial N−1 dimensions. The test arrays are Hamming distances on ${\left\{0,1\right\}}^{n}$, leaf-to-leaf path lengths on a binary tree, shortest paths on a cycle, and a random symmetric array (Data Availability). Where the array has real structure the rank finds it: for Hamming distance on ${\left\{0,1\right\}}^{n}$ the positive spectrum is exactly *n* eigenvalues, all equal to $n\;2^{n-2}$, and replacing σ by $\sqrt{\sigma }$ (the $L^{1}\to L^{2}$ embedding) gives B⪰0 with rank exactly *n*, the number of bits.

### 5.2. The Emergent Distance Is a Metric, and When It Is Not

In length units (Section 5.3) write the kernel as $\rho =f(d^{2})$ with

(10) $$f\left(q\right)=\frac{\lambda^{2}}{q+\lambda^{2}}=\int_{0}^{\infty }e^{-qs}\;\lambda^{2}e^{-\lambda^{2}s}\;ds,\;q\geq 0.$$

*f* is completely monotone, and by Bernstein’s theorem [38] it is a Laplace transform of a non-negative measure; the measure is exponential, the same law the maximum-entropy step produced.

Proposition.

Let ρ be symmetric with $\rho_{ii}=1$, $\rho_{ij}\in \left(0,1\right]$, $d_{ij}=\lambda \sqrt{\rho_{ij}^{-1}-1}$, and $B=-\frac{1}{2}P_{c}D^{\left(2\right)}P_{c}$ with $D_{ij}^{\left(2\right)}=d_{ij}^{2}$. Then (i) $d_{ij}=d_{ji}\geq 0$, with equality if and only if $\rho_{ij}=1$; (ii) *d* is Euclidean, hence satisfies the triangle inequality, if and only if B⪰0 [37], with embedding dimension rankB; (iii) if $\rho_{ij}=f(∥x_{i}-x_{j}{∥}^{2})$ with $x_{i}\in R^{n}$, then B⪰0, $d_{ij}=∥x_{i}-x_{j}∥$, and rankB=n; (iv) since *f* is completely monotone, $f(∥x-y{∥}^{2})$ is positive definite on every $R^{n}$ [39]. Items (i)–(iii) use only strict monotonicity of *f*.

When the construction fails.

Take $N=L^{2}$ patches on a square lattice of spacing *a*, with x=λ/a, the setting of Section 7 and Section 8. On the 4×4 lattice at $x=\sqrt{2}$ with open (straight) separations, minspecB=0, rankB=2, and minspecρ=+0.071. With periodic (minimum-image) separations, $\min\mathrm{spec}B=-4a^{2}$, independent of *x* because the inversion returns the minimum-image separations for every λ, and minspecρ=−0.133. The minimum-image distance is a metric, but the flat torus does not embed isometrically in any Euclidean space. The negative eigenvalue of ρ is categorical: for unit-variance variables $s_{i}$ with correlations $\rho_{ij}$, $v^{⊤}\rho \;v$ is the variance of $\sum_{i}v_{i}s_{i}$, so the periodic target (the correlation matrix the model is asked to reproduce) is not the correlation matrix of any random variables. It is admissible for x<1.2100 and inadmissible above it (Section 8.4).

**Figure 2:**
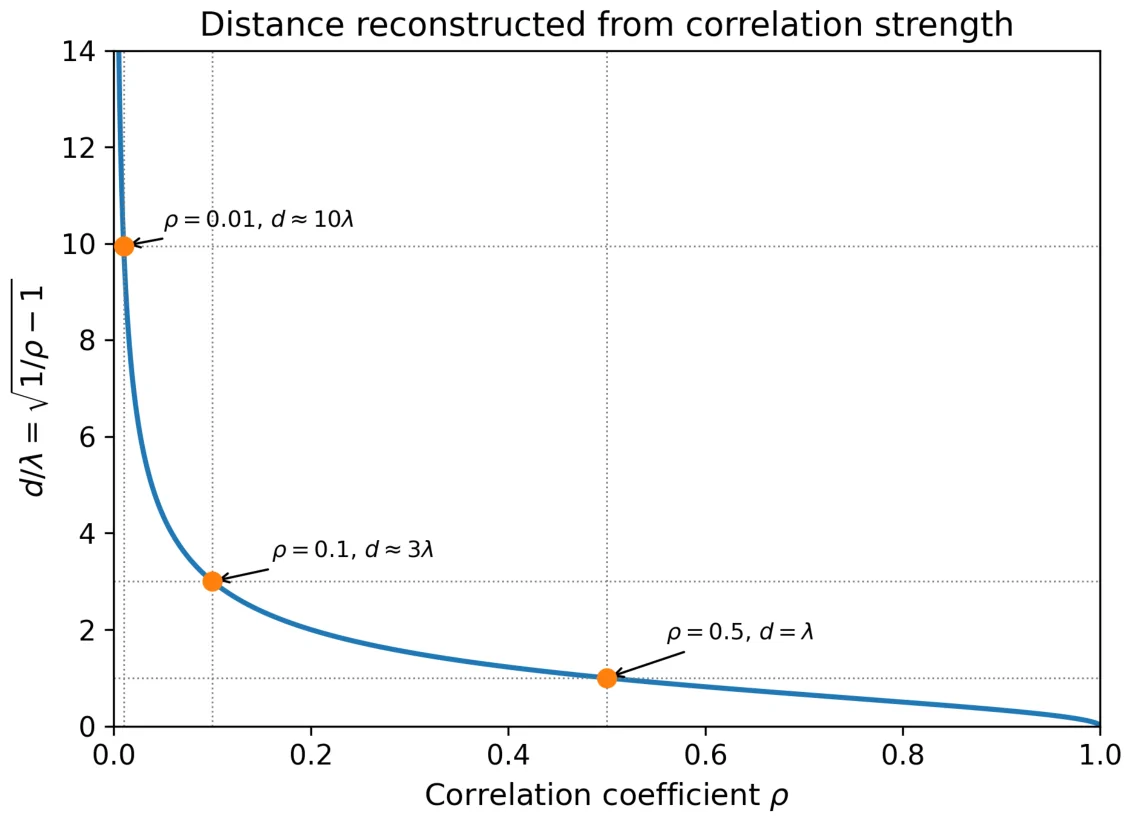
Emergent distance $d/\lambda =\sqrt{1/\rho -1}$ against correlation ρ: ρ=0.5 gives d=λ, ρ=0.1 gives d≈3λ, and ρ=0.01 gives d≈10λ.

The two conditions are ordered: B⪰0 implies ρ⪰0 by (iv), and the converse fails. On a sphere with geodesic separations *B* is already non-Euclidean at N=30, while ρ stays positive until N≈500 (Appendix B); B⪰0 is the stronger test. So the inversion is safe on any screen that embeds in Euclidean space, and test (ii) flags every screen that does not. Figure 2 shows the inversion.

### 5.3. Signature, Invariant Speed, and Length Unit

The kernel is even in σ, so the reconstruction returns a Euclidean configuration with no orientation and no causal structure. These come from the finite-invariant-speed postulate. A density quadratic in two derivatives has a characteristic speed; sound is an example, with a speed tied to the medium. Possibility space has no medium, so the speed is the same in every frame. With homogeneity and the group property, frame independence then leaves Lorentzian, Galilean, and Euclidean kinematics [40]; causal order excludes the third, and a finite invariant speed, the postulate of Table 1, selects the first. The reconstruction describes a spacelike slice of the resulting cone. Measured in the unit of σ, the invariant speed is

(11) v=1,

and it is this postulate that puts the relative minus sign in the field theory of Appendix C.3. The finiteness of the speed is assumed.

Expressing the reconstructed coordinates as lengths requires one velocity, and the invariant speed is the one the model has. Writing it as *c*,

(12) $$d_{ij}=c\;\sigma_{ij},\;\lambda =c\;\lambda_{\sigma }=\frac{c}{\Omega },$$

so that $\sigma_{ij}=d_{ij}/c$ defines the length $d_{ij}$. The conversion must be linear, and the reason uses the array alone. A monotone reparametrization *h* of σ with h(0)=0 that maps every collinear (rank-one) configuration to a collinear one satisfies h(s+t)=h(s)+h(t) on the points 0, *s*, s+t, and is therefore linear. Nonlinear choices fail visibly: $\sqrt{\sigma }$ raises rankB to N−1, and $\sigma^{2}$ violates B⪰0. For a locally flat region with $d_{ij}=r$,

(13) $$\rho \left(r\right)=\frac{\lambda^{2}}{r^{2}+\lambda^{2}}.$$

The content of (12) is a choice of unit, the same choice made when the meter is defined from *c* [41]. Only dimensionless ratios carry physical content.

### 5.4. Realizability

That the kernel on a Euclidean configuration gives B⪰0 is a theorem; that the correlations of a physical horizon satisfy it is a local, falsifiable claim. It is the point at which the construction meets data.

## 6. Dynamics on the Reconstructed Screen

Section 2, Section 3, Section 4 and Section 5 give a static correlation structure on a reconstructed screen. This section asks how that structure responds when the mean rate Ω varies from patch to patch. It uses the Fisher metric of the exponential family [42,43], the locality postulate, and the invariant speed of Section 5.3, and it ends with the form of the Einstein equation.

### 6.1. Fisher Metric and Shift Symmetry

For p(ω;Ω), $\partial_{\Omega }\log p=-1/\Omega +\omega /\Omega^{2}$, so the Fisher information is $I\left(\Omega \right)=1/\Omega^{2}$ and

(14) $$ds_{F}^{2}=\frac{d\Omega^{2}}{\Omega^{2}}=d\varphi^{2},\;\varphi \equiv \log\Omega .$$

φ is the affine coordinate, and the rescaling $\Omega \mapsto e^{C}\Omega$, that is φ→φ+C, preserves the metric: only ratios of rates carry statistical meaning. On the screen Σ of Section 5, with positions *x* and spacing *a*, the rate may vary, Ω(x)=c/λ(x) and φ(x)=logΩ(x). Letting each patch have its own mean rate is still maximum entropy and adds no assumption; treating the values as samples of a differentiable field is the locality assumption (Table 1).

### 6.2. From Pair Divergence to a Local Action

A gradient action for φ should measure how distinguishable neighboring patches are. The kernel between two patches with different rates (Appendix C.4) cannot serve: its expansion in Δφ has coefficients that depend on σ and on the mean $\bar{\varphi }=\left(\varphi_{i}+\varphi_{j}\right)/2$, so it breaks the shift symmetry. The divergence between the two rate densities does not.

Step 1: the divergence of two patches.

Two patches with mean rates $\Omega_{i},\Omega_{j}$ differ by $\Delta \varphi =\ln(\Omega_{i}/\Omega_{j})$. The Kullback–Leibler divergence of two densities is $D_{\mathrm{KL}}\left(p∥q\right)=\int p\ln\left(p/q\right)$; for the two exponentials $D_{\mathrm{KL}}\left(p_{i}∥p_{j}\right)=e^{\Delta \varphi }-\Delta \varphi -1$, and the symmetrized (Jeffreys) divergence is

(15) $$J_{ij}\;=\;D_{\mathrm{KL}}\left(p_{i}∥p_{j}\right)+D_{\mathrm{KL}}\left(p_{j}∥p_{i}\right)\;=\;2\left(\cosh\Delta \varphi -1\right)\;=\;{\left(\Delta \varphi \right)}^{2}+\frac{1}{12}{\left(\Delta \varphi \right)}^{4}+\ldots$$

It depends on Δφ alone, with no σ and no $\bar{\varphi }$, so it is invariant under φ→φ+C to all orders, and its leading coefficient is 1, the Fisher metric (14). (The script J keeps it apart from the Ising couplings $J_{ij}$).

Step 2: the continuum.

Take the screen to be a spatial slice reconstructed from bulk patches, with rankB=D=3 (for a horizon cross-section D=2). Summing over nearest neighbors and writing $\Delta \varphi =a\;\partial_{\mu }\varphi$,

(16) $$\frac{1}{2}\;\;\sum_{\langle ij\rangle }\;J_{ij}\;⟶\;\frac{1}{2}\;a^{2-D}\;\int \;d^{D}x\;{\left(\nabla \varphi \right)}^{2}.$$

Two assumptions enter: the sum runs over neighbors only, and φ is differentiable (Table 1). A weighting of the neighbor sum multiplies a σ-free quantity, so it keeps the shift symmetry. Equation (16) fixes the relative weight of the gradient terms through the Fisher metric; the overall constant, $a^{2-D}$ here, is replaced by the decay constant $f^{2}$ of Section 6.4. The divergence gives only the spatial term ${\left(\nabla \varphi \right)}^{2}$, with positive sign. The invariant-speed postulate adds the time direction and completes it to $g^{\mu \nu }\partial_{\mu }\varphi \;\partial_{\nu }\varphi$, with the time term of opposite sign.

### 6.3. No Potential; Truncation

A function with F(φ)=F(φ+C) for all *C* is constant, so a potential V(φ), a mass term, or a coupling $\xi R\varphi^{2}$ is excluded within the Fisher sector. The argument constrains only the zero-derivative sector. Any absolute scale breaks the symmetry: the patch spacing *a*, or *G* through $a=\ell_{P}$. The largest candidate for an induced potential is a vacuum energy of the correlations at the local scale, of order $\hbar c/\lambda {\left(x\right)}^{4}\propto e^{4\varphi }$. It would drive φ directly, as a tadpole and not only as a mass, and at wavelength *L* it would exceed the gradient energy by a factor of order ${\left(L/\lambda \right)}^{2}$. Whether such a term arises is open. The field equation below is conditional on its absence (Table 1). If *a* is an absolute scale against which λ(x) varies, this requires the cancellation behind the undetermined Λ to hold at every value of φ. If *a* and every other dimensionful scale, the masses of matter included, track λ(x), the term is a constant in the frame of matter’s clocks and rulers and contributes only to Λ. The construction does not decide between these readings. The potential *V* of Section 4 belongs to the kernel’s variational description and is not a term in the Fisher action.

With signature (−,+,+,+) the Fisher action is

(17) $$S_{F}\left[\varphi ,g\right]=-\frac{1}{2}\int g^{\mu \nu }\;\partial_{\mu }\varphi \;\partial_{\nu }\varphi \;\sqrt{-g}\;d^{4}x,$$

a massless shift-symmetric scalar with □φ=0. Shift symmetry also permits ${\left(\partial \varphi \right)}^{4}$ and $\xi R{\left(\partial \varphi \right)}^{2}$; dropping them is an effective-field-theory truncation, with the cutoff at the scale where the metric description fails (Section 9),

(18) $$M_{\mathrm{eff}}=L_{\mathrm{onset}}^{-1}=O\left(\lambda^{-1}\right).$$

The suppression $R\;L_{\mathrm{onset}}^{2}$ is $∼10^{-99}$ in the solar system, $10^{-76}$ at a solar-mass horizon, and $10^{-11}$ at $H∼10^{13}$ GeV. That the cutoff is Planckian is inherited from $a=\ell_{P}$; the truncation is an assumption.

### 6.4. Stress Tensor, Physical Units, Lovelock, and the Coupling

Varying $S_{F}$ gives the standard stress tensor of a massless scalar,

(19) $$T_{\mu \nu }^{F}=\partial_{\mu }\varphi \;\partial_{\nu }\varphi -\frac{1}{2}g_{\mu \nu }\;{\left(\partial \varphi \right)}^{2},$$

conserved on shell, with $T_{00}^{F}\geq 0$. It is built from the dimensionless φ and has units $\left[m^{-2}\right]$. Let $\lambda_{0}$ be the constant background correlation length fixed by the entropy matching of Section 7 and Section 8. The minimum energy of a mode is linear in its rate (Section 4), so a correlation quantum at that scale carries $E=\hbar \Omega =\hbar c/\lambda_{0}$ in a cell of volume $\lambda_{0}^{3}$. Since $T_{00}^{F}∼\lambda_{0}^{-2}$ at the background scale, the factor that carries $T^{F}$ to this energy density is

(20) $$T_{\mu \nu }^{\left(\varphi \right)}\;\equiv \;f^{2}\;T_{\mu \nu }^{F},\;f^{2}\;\equiv \;\frac{\hbar c}{\lambda_{0}^{2}}.$$

A field-dependent $f^{2}$ would violate $\nabla^{\mu }T_{\mu \nu }^{\left(\varphi \right)}=0$; with $\lambda_{0}$ constant, $f^{2}$ is a decay constant, and varying $S_{\varphi }=f^{2}S_{F}=-\frac{f^{2}}{2c}\;\int \;{\left(\partial \varphi \right)}^{2}\sqrt{-g}\;d^{4}x$ returns (20). Converting to physical units requires one action scale, taken to be *ℏ*, which enters through $\ell_{P}={\left(\hbar G/c^{3}\right)}^{1/2}$ when $a=\ell_{P}$. At $\lambda_{0}=\ell_{P}$, since $\hbar c/\ell_{P}^{2}=c^{4}/G$,

(21) $$T_{\mu \nu }^{\left(\varphi \right)}=\frac{c^{4}}{G}\;T_{\mu \nu }^{F}\;\left(\lambda_{0}=\ell_{P}\right).$$

That step is the definition of $\ell_{P}$ rearranged, so *G* leaves as it entered. The content is the dimensionless ratio in

(22) $$\kappa_{F}\;\equiv \;\frac{8\pi G}{c^{4}}\;f^{2}\;=\;8\pi {\left(\frac{\ell_{P}}{\lambda_{0}}\right)}^{\;2},$$

which is 14.8 at $\lambda_{G}$ and 4π at $\lambda_{\mathrm{bin}}$; the number 8π in the field equation is the standard one, and what the construction supplies is that $x=\lambda_{0}/a$ is of order one, so that $\lambda_{0}/\ell_{P}$ is of order one given $a=\ell_{P}$.

If the field back-reacts through a local covariant equation $H_{\mu \nu }=\kappa T_{\mu \nu }$, with $H_{\mu \nu }$ symmetric, divergence-free, and built from the metric and its first two derivatives, Lovelock’s theorem fixes $H_{\mu \nu }$ in four spacetime dimensions to $\alpha G_{\mu \nu }+\Lambda g_{\mu \nu }$. The dimension is the 3+1 of a bulk reconstruction with rankB=3 and the invariant-speed postulate (Table 1). So

(23) $$\begin{array}{c} G_{\mu \nu }+\Lambda \;g_{\mu \nu }=\frac{8\pi G}{c^{4}}\;T_{\mu \nu }^{\left(\varphi \right)}\;=\;\kappa_{F}\;T_{\mu \nu }^{F} \end{array}.$$

Lovelock fixes the form, not the constant, and Λ is not fixed by this argument or by any other in the paper.

Fixing κ.

For a static Fisher field, with $Q={\left|\nabla \varphi \right|}^{2}$, the energy density is $\rho_{F}=\frac{1}{2}Q$ and the isotropic pressure $p_{F}=-\frac{1}{6}Q$, so

(24) $$w=\frac{p_{F}}{\rho_{F}}=-\frac{1}{3}\;⟹\;\rho_{F}+3p_{F}\equiv 0,$$

the equation of state of a global monopole [44]. The Newtonian source vanishes, so the Newtonian limit cannot fix κ for this source alone. The Clausius relation on local Rindler horizons can [6]. There, the heat flux through the horizon is $T_{\mu \nu }k^{\mu }k^{\nu }$, which must be non-negative, and the Fisher field satisfies this identically, $T_{\mu \nu }^{F}k^{\mu }k^{\nu }={\left(k\cdot \partial \varphi \right)}^{2}\geq 0$. With δQ=TδS, S=ηA, and the Unruh temperature, Raychaudhuri’s equation gives $G_{\mu \nu }+\Lambda g_{\mu \nu }=\left(2\pi /\hbar c\;\eta \right)\;T_{\mu \nu }$, and the Bekenstein–Hawking coefficient $\eta =c^{3}/4G\hbar$ gives $\kappa =8\pi G/c^{4}$. That coefficient is the same $\frac{1}{4}$ used in the entropy matching, so no new number enters, and since the form was fixed without horizons the argument is not circular.

### 6.5. The Source as a Correlation Perturbation and Matter

The correlation field is the kernel evaluated at a fixed reference separation $\sigma_{*}$ with the local correlation length,

(25) $$\chi \left(x\right)\;\equiv \;\rho \;\left(\sigma_{*};\lambda \left(x\right)\right),$$

with constant unperturbed value $\chi_{0}=\rho \left(\sigma_{*};\lambda_{0}\right)$. Since ∂ρ/∂logλ=2ρ(1−ρ),

(26) $$\delta \chi \;=\;-2\chi_{0}\left(1-\chi_{0}\right)\;\delta \varphi ,$$

so a change of the Fisher coordinate is a change of correlation. The Jacobian is a field redefinition and leaves $T_{\mu \nu }$ unchanged. The source is built from $\partial_{\mu }\varphi$ and vanishes on a constant background.

Matter.

Ordinary matter couples through general covariance,

(27) $$G_{\mu \nu }+\Lambda \;g_{\mu \nu }=\frac{8\pi G}{c^{4}}\;\left(T_{\mu \nu }^{\left(\varphi \right)}+T_{\mu \nu }^{\mathrm{matter}}\right),$$

and with matter present the usual Newtonian limit applies.

## 7. Entropy Matching: The Gaussian Sector

Section 2, Section 3, Section 4 and Section 5 fix the form of the kernel and show that it defines a screen. What remains free is its scale λ relative to the patch spacing *a*. Take $a=\ell_{P}$ (Table 1), so a horizon of area *A* holds $N=A/\ell_{P}^{2}$ patches, and ask which λ lets the kernel’s correlations supply the deficit $c_{\mathrm{total}}$. The deficit may be read as total correlation, redundancy, or a reduction of the Hilbert-space dimension below a naive tensor product. To turn the kernel into an entropy one needs random variables with these correlations. The kernel fixes only second moments, and the maximum-entropy law with given second moments is Gaussian for continuous variables (this section) and the pairwise Ising model for binary ones (Section 8).

A pairwise mutual-information sum, with $I_{\mathrm{pair}}\left(\rho \right)=-\frac{1}{2}\log_{2}\left(1-\rho^{2}\right)$ (Appendix D), gives a first estimate. Written as a continuum integral with cutoff r=a it matches at λ/a≃1.025, and the direct lattice sum matches at 0.898; the estimate depends on the cutoff, and it overcounts information shared by three or more patches. It serves only as a comparison. The Gaussian calculation below has neither problem.

### 7.1. The Gaussian Covariance Calculation

For a zero-mean Gaussian field with unit variances and correlation matrix $K_{ij}=\lambda^{2}/\left(d_{ij}^{2}+\lambda^{2}\right)$, the total correlation per patch is

(28) $$\begin{array}{c} c_{G}=-\frac{1}{2N}\log_{2}\det K \end{array},$$

exact for Gaussian statistics and including all orders. On an L×L periodic screen *K* is block-circulant, and with x=λ/a, $c_{G}\left(x;L\right)=-\frac{1}{2L^{2}}\sum_{p,q}\log_{2}\eta_{pq}$ over its DFT eigenvalues $\eta_{pq}$. The $1/r^{2}$ tail makes $\sum_{j}K_{ij}∼\ln L$, but that is a single eigenvalue, and its effect on $c_{G}$ is $O(L^{-2}\ln\ln L)$. Positivity of the $\eta_{pq}$ is not automatic on a torus; it holds at the scales used here (Appendix B). The results are

(29) $$c_{G}\left(1\right)≃0.362656,\;c_{G}\left(x_{G}\right)=c_{\mathrm{total}}\;\mathrm{at}\;\begin{array}{c} \frac{\lambda_{G}}{a}≃1.302 \end{array}.$$

**Figure 3:**
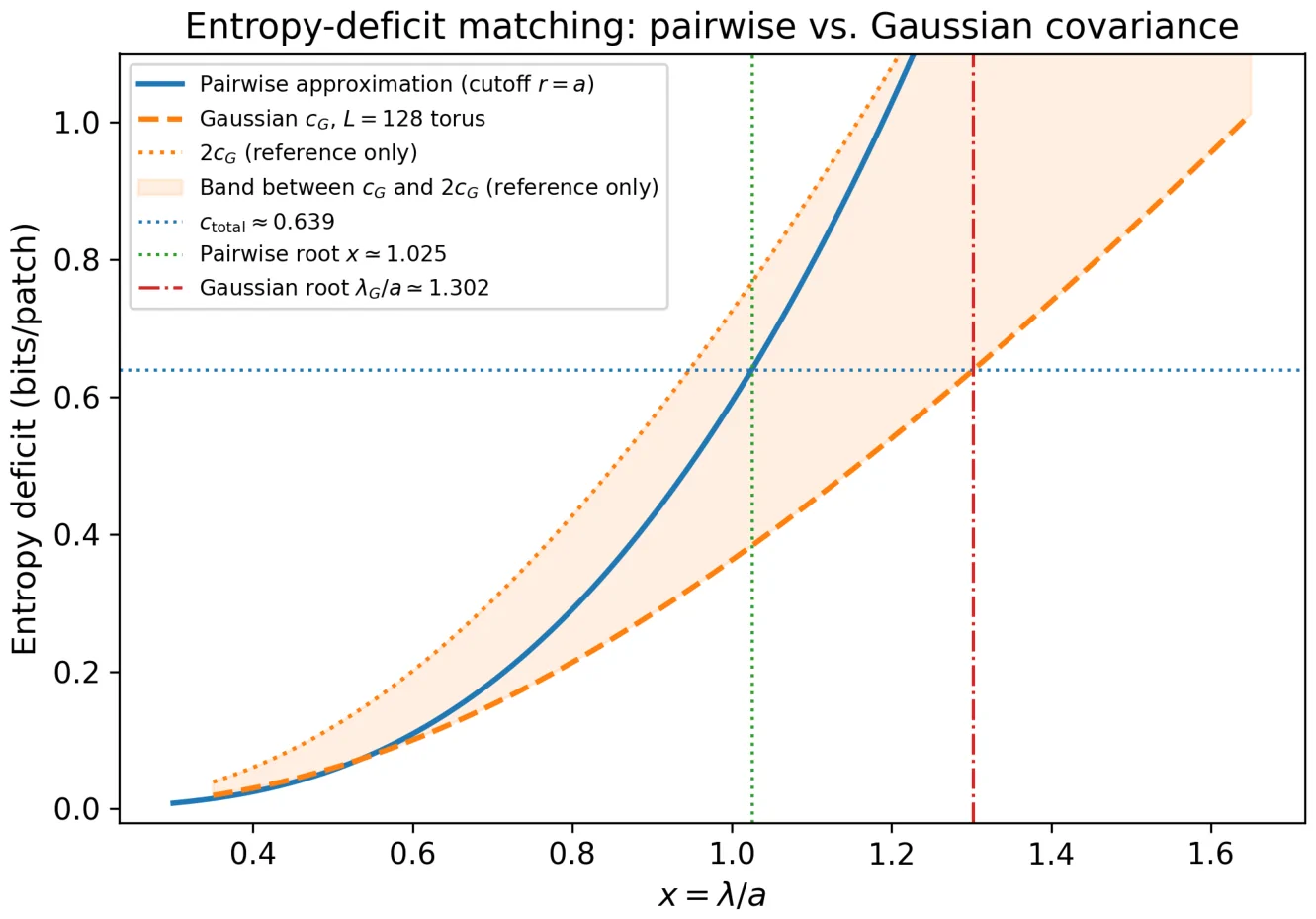
Entropy-deficit matching against x=λ/a. Solid: pairwise approximation (continuum integral with cutoff r=a). Dashed: Gaussian $c_{G}$ (L=128 torus). Dotted and shaded: the curve $2c_{G}$ and the band between $c_{G}$ and $2c_{G}$, shown for reference; no quantum bound is derived here. Horizontal: $c_{\mathrm{total}}\approx 0.639$. Vertical: the pairwise root x≃1.025 and the Gaussian root $\lambda_{G}/a≃1.302$.

This is a calibration: one number fitted to one empirical input (Figure 3). That the root is Planckian, and not many orders of magnitude away, is the content. The binary results of Section 8 use $\lambda_{\mathrm{bin}}=\sqrt{2}\;a$ instead.

### 7.2. Sensitivity to the Scale

Imposing $\lambda =\ell_{P}$ instead of solving for λ leaves

(30) $$c_{\mathrm{total}}-c_{G}\left(\ell_{P}\right)\;≃\;0.2767$$

bits per patch, but that residual is set by the stipulated scale and changes sign across the Planckian range (Table 4); the paper makes no quantitative use of it. What survives is qualitative. The Gaussian two-point sector supplies between about half and somewhat more than all of the deficit across the Planckian range, and its slope at the root is $dc_{G}/dx≃1.0$, so a residual δc shifts the root by δx≈δc. Since *x* is uncertain by order one across that range, any residual attributed to multipartite correlations is smaller than the uncertainty in λ.

## 8. Binary Patch Model

The Gaussian calculation treats patch variables as continuous, which overstates the multi-information of a Planck-area patch that carries at most one bit. This section assigns each patch a binary state $s_{i}\in \left\{-1,+1\right\}$, the simplest discrete degree of freedom (any *q*-state variable is $\left\lceil \log_{2}q\right\rceil$ binary ones). The $s_{i}$ are a second, coarse-grained description of each patch; only their pairwise correlations are taken from the kernel, $K_{ij}=\rho \left(d_{ij}\right)$.

Throughout this section the patches sit on an L×L square lattice with spacing *a*; $d_{ij}$ is the separation of lattice sites *i* and *j*, and ρ is evaluated at those separations. No continuum limit is taken, except the L→∞ extrapolation of Section 8.6. All values refer to a two-dimensional screen (rankB=2), appropriate for horizons; bulk patches are a separate calculation.

Two boundary conventions are used and marked by superscripts. Periodic lattices ($c^{\mathrm{per}}$, minimum-image separations) match the Gaussian torus calculation and are used in Section 8.4 and Section 8.5. Open lattices ($c^{\mathrm{open}}$, straight separations) give a monotone sequence of lower bounds and are used for the L→∞ limit in Section 8.6. At L=5 they differ by about 30% in the summed pair correlation, so finite-*L* values are never compared across conventions; the two share one limit, because boundary effects are O(1/L) per site (Appendix E).

### 8.1. The Pairwise Maximum-Entropy Ising Model

The maximum-entropy distribution on ${\left\{\pm 1\right\}}^{N}$ with prescribed $\langle s_{i}s_{j}\rangle =K_{ij}$ is

(31) $$P\left(s\right)=\frac{1}{Z}\exp\;\left(\sum_{i<j}J_{ij}\;s_{i}s_{j}\right),$$

with $J_{ij}$ fixed by the convex dual $\nabla_{J}\left[\log Z\left(J\right)-\sum_{i<j}J_{ij}K_{ij}\right]=0$. For one isolated pair $\langle s_{1}s_{2}\rangle =\tanh J$, so $J=\mathrm{artanh}\rho (d_{12})$ with $d_{12}$ the lattice separation; for N>2 there is no closed form and the $J_{ij}$ are fitted numerically. Whether the fitted couplings are long-range in the sense of [45,46,47] is not established (Section 8.6); Onsager’s nearest-neighbor solution does not apply. The multi-information per patch is $c_{\mathrm{Ising}}=1-H_{\mathrm{Ising}}/N$, with $H_{\mathrm{Ising}}$ the joint Shannon entropy in bits. $c_{\mathrm{Ising}}\leq c_{G}$ holds at every (L,x) computed here; it is an observation, since the two are functionals of different measures.

### 8.2. A Structural Identity Connecting Pair Interactions to Mutual Information

With f(J)=JtanhJ−logcoshJ,

(32) $$f\left(\mathrm{artanh}K\right)=I_{B}\left(K\right)=\frac{1+K}{2}\ln\left(1+K\right)+\frac{1-K}{2}\ln\left(1-K\right),$$

the binary mutual information of two ±1 variables with correlation *K*, in nats. Hence, in bits,

(33) $$c_{\mathrm{Ising}}=\underset{c_{\mathrm{Bethe}}}{\underset{︸}{\frac{1}{N\ln2}\sum_{i<j}I_{B}\left(K_{ij}\right)}}\;-\;\left(\mathrm{loop}\;\mathrm{corrections}\right),$$

where $c_{\mathrm{Bethe}}$ is the Bethe (tree) approximation and the loop terms remove information counted twice around cycles. They are negative in every case computed here, but on frustrated graphs the sign is not guaranteed, so (33) is a decomposition and not a bound.

### 8.3. The $\sqrt{2}$ Scale: The Edge of Binary Realizability

Binary patches cannot carry an arbitrary correlation length. For ±1 variables with $K_{ij}=E\left[s_{i}s_{j}\right]$, under any joint law, a classical triangle inequality for binary random fields holds [48],

(34) $$1-K_{ij}-K_{jk}+K_{ik}=4\mathrm{Pr}\;\left(s_{j}\neq s_{i},\;s_{j}\neq s_{k}\right)\geq 0.$$

For three collinear patches at spacings *a*, *a*, and 2a, with $t=a^{2}/\lambda^{2}$, the kernel gives

(35) $$1-2K\left(a\right)+K\left(2a\right)=\frac{2t(2t-1)}{\left(1+t)(1+4t\right)},$$

which is positive for $\lambda <\sqrt{2}\;a$, vanishes at $\lambda =\sqrt{2}\;a$, and is negative above. So no correlation length $\lambda >\sqrt{2}\;a$ can be carried by binary patches on a lattice of spacing *a*, on the square lattice or any other that contains a straight row of three sites. Conversely, exact linear-programming membership in the cut polytope [49] holds up to $\lambda =\sqrt{2}\;a$ on every finite cluster tested (chains, 3×3, and open 4×4 blocks), and at $\lambda =\sqrt{2}\;a$ exact maximum-entropy laws carrying every pair correlation exist on the open 6×6 lattice and the 7×7 torus, so no other inequality binds first there; realizability on the infinite lattice is not proved. Because the kernel is smooth at the origin, no continuum binary field realizes it for any λ [48], so a lattice scale is required. $\lambda_{\mathrm{bin}}=\sqrt{2}\;a$ is therefore the largest correlation scale binary patches can realize, and it is fixed before any entropy comparison, so comparing $c_{\mathrm{Ising}}$ with $c_{\mathrm{total}}$ there is not circular. Setting λ exactly at the edge remains a modeling choice (maximal coherence). At the edge the maximum-entropy law gives zero weight to a patch that disagrees with both collinear neighbors along a row or a column, so every admissible law lives on the configurations without such a patch. Pair couplings fitted over all configurations grow without bound as the edge is approached (max|J| rises from 1.9 at λ=1.41a to 8.0 at $\sqrt{2}\;a$ on the open 3×3 lattice), which is how a pairwise model emulates this hard constraint; on the restricted support the remaining couplings are finite (max|θ|=0.63 on the open 6×6 lattice). The maximum entropy is continuous on the closed polytope, so the multi-informations below are well defined. At this scale every one of a patch’s eight cell neighbors has $K\geq \frac{1}{2}$ (K(a)=2/3, $K(a\sqrt{2})=1/2$). $\lambda_{G}≃1.302\;a$, by contrast, is solved for.

### 8.4. The Multi-Information at $\lambda_{\mathrm{bin}}$

**Figure 4:**
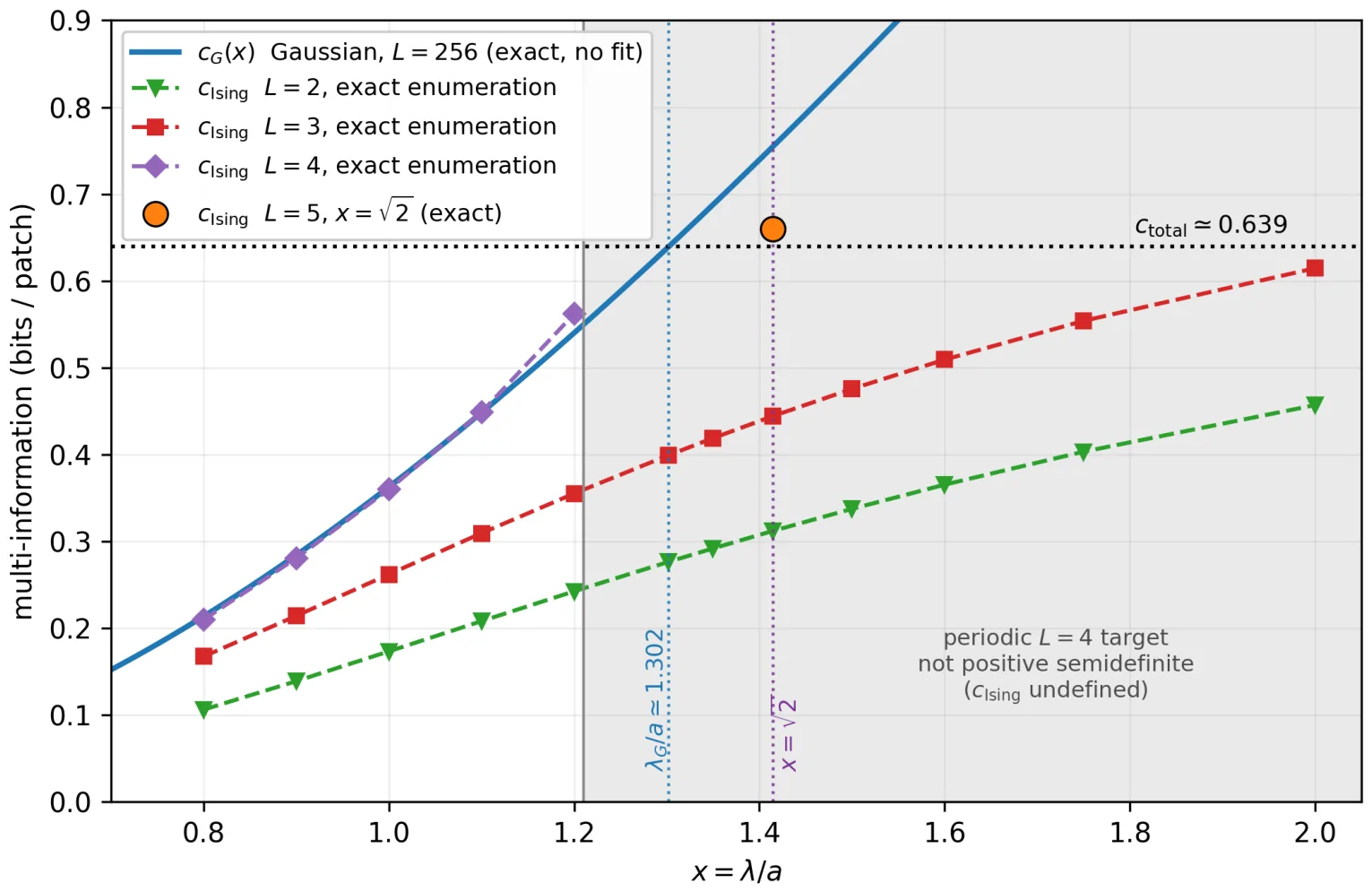
Multi-information per patch against x=λ/a on a periodic lattice. $c_{G}$ by FFT on L=256; binary points at L=2, 3, and 4 by exact enumeration (residuals $10^{-13}$ to $10^{-6}$); the marker is $c_{\mathrm{Ising}}^{\mathrm{per}}\left(5,\sqrt{2}\right)=0.6601$. Shaded: $x>x_{c}=1.2100$, where the periodic L=4 target is not positive semidefinite and $c_{\mathrm{Ising}}$ is undefined. Dotted: $c_{\mathrm{total}}$. Vertical: $\lambda_{G}/a$ and $\sqrt{2}$.

At this scale the multi-information can be compared with $c_{\mathrm{total}}$. Figure 4 shows $c_{G}\left(x\right)$ and $c_{\mathrm{Ising}}^{\mathrm{per}}\left(x\right)$ at L=2, 3, and 4; binary values lie below the Gaussian curve throughout. Two facts limit what can be said at $x=\sqrt{2}$. The periodic L=4 target there has smallest eigenvalue −0.1333, so it is not a correlation matrix and $c_{\mathrm{Ising}}\left(4,\sqrt{2}\right)$ does not exist (Section 5.2). The lattice size is then a choice; L=5 is used because it is the largest lattice on which the partition function can be enumerated exactly ($2^{25}$ states), which checks the computation on the support of Section 8.3. There,

(36) $$c_{\mathrm{Ising}}^{\mathrm{per}}\left(5,\sqrt{2}\right)=0.6601,$$

with maximum correlation residual $2\times 10^{-14}$ on the support ($1.7\times 10^{-4}$ for the fit over all $2^{25}$ states). It exceeds $c_{\mathrm{total}}$ by 3.2%; equivalently the residual entropy density, 0.340 bits per site, lies 5.8% below the Bekenstein–Hawking

(37) $$\frac{1}{4\ln2}\approx 0.361\;\mathrm{bits}\;\mathrm{per}\;\mathrm{site}=\frac{1}{4}\;\mathrm{nat}\;\mathrm{per}\;\mathrm{site}.$$

Because *L* is chosen, this is an order-of-magnitude check; the claim about the thermodynamic limit is the subject of Section 8.6. $c_{\mathrm{Ising}}$ is not scale-invariant: a multi-information per site changes when a fixed field is sampled more finely.

### 8.5. Is the Agreement Determined or Fitted?

With a kernel, a lattice, *x*, and *L* to choose, one might hit any target. Two checks bear on this. First, the Gaussian matching is stiff: at the root

(38) $$x_{G}=1.3021,\;{\left.\frac{dc_{G}}{dx}\right|}_{x_{G}}=0.999\;\mathrm{bits}\;\mathrm{per}\;\mathrm{unit}\;x,$$

so matching $c_{\mathrm{total}}$ to 1% needs *x* to 0.5%; $c_{G}$ is −43%, +18%, and +123% off at x=1, $\sqrt{2}$, and 2. Second, the ratio of the binary to the Gaussian multi-information at fixed *x* is predicted. Weighting the single-pair ratio $I_{B}/I_{G}$, both in bits with $I_{G}=-\frac{1}{2}\log_{2}\left(1-K^{2}\right)$, over the pairs of the periodic L=5 lattice at $x=\sqrt{2}$ gives

(39) $${\langle \frac{I_{B}}{I_{G}}\rangle }_{\mathrm{per}}=0.885,$$

against the observed $c_{\mathrm{Ising}}^{\mathrm{per}}\left(5,\sqrt{2}\right)/c_{G}\left(\sqrt{2}\right)=0.6601/0.7543=0.875$, a 1.1% agreement on the same convention at L=5. In the limit the observed ratio is $c_{\infty }/c_{G}\left(\sqrt{2}\right)=0.847$, so this agreement is specific to the lattice size. The first check shows that *x* was not tuned to hit $c_{\mathrm{total}}$; the choice of *L* remains.

### 8.6. The Thermodynamic Limit $c_{\infty }$ and 1−1/(4ln2)

Does $c_{\mathrm{Ising}}\left(L,\sqrt{2}\right)$ converge to *exactly* 1−1/(4ln2) as L→∞? I have no proof. The evidence and its limits are summarized here; methods, fits, and controls are in Appendix E.

The kernel falls as $\rho ∼d^{-2}$, so on a two-dimensional screen the correlation accumulated by one patch out to separation *L* grows as lnL: the marginal case, reached with no parameter adjusted. The open-boundary sequence (A13), exact for L=2–7, rises monotonically. By subadditivity of entropy each entry is a rigorous lower bound on $c_{\infty }$ (Appendix E). Because every admissible law at $\lambda_{\mathrm{bin}}$ lives on the hard-constraint support of Section 8.3, transfer matrices restricted to that support are far smaller, and two bounds follow. On open strips, with no further assumption,

(40) $$c_{\infty }\;\geq \;0.6135,$$

4.0% below 1−1/(4ln2). On periodic cylinders, with every separation of up to four spacings in each direction constrained individually,

(41) $$c_{\infty }\;\geq \;0.6389,$$

a gap of 0.07%. Equation (41) rests on one unproved step: that at a fixed set of constraints the cylinder’s entropy per site converges to the plane’s. A pre-registered test extended the cylinders to circumferences 13–17 and found the plane values within $7\times 10^{-5}$ of the cylinder values at circumferences 10–12, for every convergence form tested (Appendix E); that supports the step within the tested forms without proving it.

Neither bound decides whether the identity is exact: both are lower bounds, and the correlations left unconstrained in (41) can still raise *c*. A test fixed in advance measures how much (Appendix E). The ratio of binary to Gaussian increments, measured on constraint sets up to four spacings, predicted the increments out to five spacings; the measured ratio, 0.86, fell inside the pre-registered band. A second pre-registered test measured the cylinder-to-plane correction directly, $+2.5\times 10^{-5}\pm 7.7\times 10^{-5}$. Together they give

(42) $$c_{\infty }\;=\;0.63906\pm 0.00008,$$

$2.6\times 10^{-4}$ below 1−1/(4ln2). So $c_{\infty }$ agrees with 1−1/(4ln2) to 0.05%. Reaching it exactly would require either correlations beyond five spacings contributing about seven times the Gaussian amount, against binary-to-Gaussian ratios of 0.84–1.23 at every earlier step and 0.86 in the test, or a width correction that converges too slowly to appear by circumference 17. Exactness is therefore not supported, though calculations of this kind cannot decide it.

An independent check agrees: the exact open value at L=7, predicted in advance from L=3–6, matched to $10^{-6}$, and the refit including it gives $c_{\infty }=0.6390\pm 0.0003$.

What can be stated positively: an exact monotone sequence, a rigorous bound, a conditional bound, and a tested extrapolation place $c_{\infty }$ within 0.05% of 1−1/(4ln2), and none of them was tuned to it. Why the agreement is so close is open, and no quantitative conclusion elsewhere in the paper depends on it. A proof would require the free energy of the two-dimensional Ising model with couplings decaying as $d^{-2}$, the marginal case in which the decay exponent equals the lattice dimension; no closed form is known.

## 9. Domain of the Metric Description

The reconstruction of Section 5 needs enough patches to fix their separations. This section finds the smallest domain for which that holds, and then extends the flat-region kernel to curved screens. Lengths are in units of λ.

### 9.1. Onset Scale

Noise gives no bound.

For one pair at separation σ the observed coherence $X_{ij}=\cos\left(\left(\omega_{i}-\omega_{j}\right)\sigma \right)$ has mean ρ(σ) and variance

(43) $$\mathrm{Var}\left[X_{ij}\left(\sigma \right)\right]=\frac{1}{2}\left(1+\rho (2\sigma )\right)-\rho^{2}\left(\sigma \right),$$

which is 0.35 at $\sigma =\lambda_{\sigma }$. At small y=Ωσ the signal $1-\rho ≃y^{2}$ and the standard deviation of one comparison, $\sqrt{5}\;y^{2}$, shrink together, so the signal-to-noise ratio of a comparison tends to the constant $1/\sqrt{5}$. Equivalently, θ=Δωσ is Laplace with scale *y*, whose Fisher information is

(44) $$I\left(y\right)=\frac{1}{y^{2}},$$

so the relative precision on a separation after *n* comparisons, $\Delta y/y\geq 1/\sqrt{n}$, is independent of *y*. Short separations are no harder to resolve than long ones.

Counting gives one.

A *D*-dimensional configuration of *N* patches has $DN-\frac{1}{2}D\left(D+1\right)$ coordinates, to be fixed by $\frac{1}{2}N\left(N-1\right)$ separations, which needs N≥D+1. A domain of size *ℓ* holds ${\left(\ell /a\right)}^{D}$ patches, so the minimum domain size is O(a); with $a=\ell_{P}$ and λ/a=O(1) (Section 7 and Section 8),

(45) $$L_{\mathrm{onset}}=O\left(\lambda \right)>0.$$

The coefficient depends on the precision demanded and is not determined here. The onset follows from the identification $a=\ell_{P}$ and would vanish as a→0 at fixed λ: the model inherits a minimum length and does not derive one. Below $L_{\mathrm{onset}}$ the description is pre-metric correlation structure.

### 9.2. Curved Screens and Cosmology

The flat-region kernel $\rho \left(r\right)=\lambda^{2}/\left(r^{2}+\lambda^{2}\right)$ is a local limit. On a spacelike hypersurface, horizon cross-section, or holographic screen it becomes

(46) $$\rho \left(x,y\right)=\frac{\lambda^{2}}{\lambda^{2}+d_{\Sigma }{\left(x,y\right)}^{2}},$$

with $d_{\Sigma }$ a separation on that surface. Geodesic separations on a curved screen fail B⪰0, while chordal ones give rankB=3 (Section 5.2). The paper therefore uses the flat-patch reading: the construction is restricted to domains small against the curvature radius $L_{c}$, where geodesic and chordal separations agree to $O({\left(L/L_{c}\right)}^{2})$. This requires $\ell_{P}\ll L_{c}$, with curvature corrections of order ${\left(\ell_{P}/L_{c}\right)}^{2}$. The natural domains are local Minkowski limits, FLRW cosmology, de Sitter-like horizons, and black-hole horizon cross-sections, none of which requires anti-de Sitter asymptotics.

As the Hubble radius approaches the Planck scale the correlation length becomes comparable to the horizon, and a causal domain of Planck radius has information capacity $I_{\mathrm{dS}}\left(R=\ell_{P}\right)=\pi /\ln2≃4.53$ bits. A smooth FLRW metric is admissible only for $L>L_{\mathrm{onset}}$, so the formal singularity at vanishing scale factor lies outside the metric-admissible sector. This is kinematic: a dynamical bounce would require a modified Friedmann equation for the pre-metric regime, which the model does not provide.

## 10. Entropy Ordering and Time Orientation

This section uses the reconstructed screens of Section 5 and the onset scale of Section 9. The construction has no background time: the comparison interval σ has no temporal order, since the kernel is even in it. A thermodynamic orientation can nevertheless be recovered. Consider the family of closed reconstructed screens at fixed λ, and let *R* be the radius of a screen, $4\pi R^{2}$ its area. Order the family by accessible holographic information, $S=\pi R^{2}/\ell_{P}^{2}$. The mean correlation $\langle \rho_{ij}\rangle$ falls, and the mean emergent distance and the screen area grow, monotonically in *R* along the family, so all of these orderings agree; they agree because each is a function of *R*, and the substantive property of the ordering is that it has a first element, since reconstruction fails below $L_{\mathrm{onset}}$. Identifying the direction of increasing *S* with the thermodynamic future orients the time coordinate τ.

The ordering supplies an orientation only. It carries no metric and no rate, so nothing in it fixes how much time separates two screens: the metric and unit of τ come from the invariant-speed postulate (Section 5.3). The causal order in that postulate is symmetric under a global time reversal, so the two inputs do not overlap: the postulate supplies the cone, and the ordering supplies which half of it is the future. The first element does not explain why the universe began near it, since the same counting makes a random geometry unlikely to sit at the first element. The construction relocates the Past Hypothesis, and it inherits its first element from $a=\ell_{P}$. A fuller treatment is developed in separate work.

## 11. Discussion

Table 1 lists the inputs. Four limitations do not appear there.

(i) The possibility space P is not derived from a microscopic theory.
(ii) The ensemble is classical: ρ is a classical average, and the Gaussian total correlation is not a von Neumann entropy. Whether a quantum state can reproduce the same correlations without detectable decoherence depends on microphysics the model does not specify; a quantized phase-rate ensemble would be needed.
(iii) The residual $c_{\mathrm{total}}-c_{G}$ is not scale-robust (Table 4), so the size of any multipartite contribution is uncontrolled.
(iv) The pairwise field theory of Appendix C.3 is promoted to *D* dimensions by analogy: its background is a zero-energy orbit, not a static solution. Nothing in the main line of the argument uses it.

Free parameters.

Of the inputs, five are dimensionless numbers that are not derived: the ratio x=λ/a, calibrated in the Gaussian sector ($x_{G}=1.302$) and chosen in the binary sector ($\sqrt{2}$); the ratio $a/\ell_{P}$, set to 1 by identification; the order-one coefficient in $f^{2}$, which the matching $T_{00}^{F}∼\lambda_{0}^{-2}$ of Section 6.4 does not fix; the evaluation scale Ωσ=1 of $\hbar_{q}$, which is unused downstream; and Λ. *G* and *ℏ* set units. The matching condition reads $\left(1-c\left(x\right)\right)\ln2\;{\left(\ell_{P}/a\right)}^{2}=\frac{1}{4}$, so the coefficient $\frac{1}{4}$ of $S_{\mathrm{BH}}$ becomes a prediction only if both *x* and $a/\ell_{P}$ are derived; deriving *x* alone predicts $a/\ell_{P}$. For comparison, the Standard Model has 19 free parameters, or 26 with massive neutrinos.

### 11.1. Why the Spatial Dimension Is Measured and Not Predicted

The kernel is a function of separation alone and cannot see the dimension; *D* enters only through the shell measure $d^{D-1}$, and maximum entropy constrains no measure. The per-shell total $\rho \left(d\right)\;\Omega_{D-1}d^{D-1}\propto d^{D-3}$ ($\Omega_{D-1}$ the area of the unit sphere) is constant exactly at D=3, but using it requires postulating its conservation. Every result here is stated for the dimension the reconstruction returns.

Number of coherence channels.

With *g* independent coherence channels, each must supply $c_{\mathrm{total}}/g$ of the deficit. For g=2 the pairwise estimate then needs λ≃0.82a (continuum integral), a correlation length below the patch spacing, where the axial neighbors fall below half correlation. A scale at or above *a* therefore favors a single effective channel, a scalar or a gauge-constrained sector.

### 11.2. Black Hole Thermodynamics

The zeroth law (constant surface gravity $\kappa_{H}$) corresponds to uniformity of the horizon patch ensemble, an equilibrium condition on the data. The first law follows from the field equation, with $S_{\mathrm{BH}}$ and the Hawking temperature entering through the Clausius relation of Section 6.4; since the coupling was fixed with the same inputs, this is a coherence check. The second and third laws are not derived.

### 11.3. Black Hole Information

The model does not address the information paradox: it has no evaporation dynamics and no Page curve [50]. Its contact with the island literature [51,52] is structural—an area term and a residual—and it cannot put a number on the split (Table 4). Nor does the inverse-square tail give Newton’s constant: ρ is a two-point function with no source, the Fisher source has $\rho_{F}+3p_{F}\equiv 0$, and a Kepler estimate $G∼c^{3}a^{2}/\hbar$ at $a=\ell_{P}$ is the definition of $\ell_{P}$ rearranged.

## 12. Conclusions

Maximum entropy over positive phase rates gives an exponential density whose characteristic function yields a nonsingular Lorentzian kernel, which is geometrically a Planck-softened inverse-square law. Given a posited separation array, classical scaling reconstructs a screen from it, with a dimension that is read off and a test that can fail. A postulated finite invariant speed supplies the Lorentzian cone. Under the locality assumption, Lovelock’s hypotheses, and a thermodynamic normalization, the Fisher geometry of the ensemble fixes the form $G_{\mu \nu }+\Lambda g_{\mu \nu }=\left(8\pi G/c^{4}\right)T_{\mu \nu }^{\left(\varphi \right)}$, with *G* an input and Λ undetermined. Of the correlation scales, the Gaussian root $\lambda_{G}≃1.302\;\ell_{P}$ is solved for; $\lambda_{\mathrm{bin}}=\sqrt{2}\;\ell_{P}$ is the largest scale that binary patches can realize, and at this scale the multi-information agrees with the Bekenstein–Hawking deficit, 0.6393, to 0.05%: the bounds are 0.6135 (rigorous) and 0.6389 (conditional), and a pre-registered extrapolation gives 0.6391. Whether the agreement is exact is open. The reconstructed screens can be ordered by accessible information, and the ordering orients time.

The framework can fail in three independent places: physical horizon data can violate B⪰0; the agreement of $c_{\infty }$ with $c_{\mathrm{total}}$ can fail if the cylinder-to-plane step does; and the field-equation chain can fail at any of its stated conditions. Either outcome is informative: confirmation would leave the five dimensionless inputs counted in Section 11 as the only adjustable steps between a maximum-entropy principle and the Bekenstein–Hawking deficit, and failure at any of these points would locate where an information-first construction breaks down.

## Figures and Tables

**Table 1:** Inputs adopted without derivation. The remaining steps follow from these; the last row collects choices that affect a single estimate, stated where they enter.

Input	Type	Role (Section)
Phase rates positive, ω≥0	postulate	makes the maximum-entropy density exponential and the kernel Lorentzian (Section 2 and Section 3)
Additivity of the constrained quantity	postulate	selects the mean as the constraint (Section 3)
Composition z(a+b)=z(a)z(b)	modeling choice	gives the oscillating comparison function (Section 2)
Coherence $\rho =E[\cos\left(\left(\omega_{i}-\omega_{j}\right)\sigma \right)]$	modeling choice	third ingredient selecting the Lorentzian (Section 4)
Separation array $\sigma_{ij}$	postulate	needed to form $\rho_{ij}$; rankB is a property of it (Section 5)
Realizability, B⪰0	falsifiable claim	required by the reconstruction; can fail (Section 5.4)
Finite invariant speed, with causal order	postulate	frame independence leaves Lorentzian, Galilean, and Euclidean kinematics; this selects the first and fixes v=1. Causal order is symmetric under time reversal; the future direction comes from Section 10 (Section 5.3)
σ=d/c	identification	converts the reconstructed configuration to lengths (Section 5.3)
Spacetime dimension 3+1	identification	taken from a bulk reconstruction with rankB=3; needed for Lovelock’s theorem (Section 6)
Locality	postulate	neighbor support and a differentiable φ; carries the two-point construction to one-point dynamics (Section 6)
No induced potential for φ	postulate	an absolute scale (*a*, or *G* via $a=\ell_{P}$) could induce one, e.g., a vacuum energy $\propto \hbar c/\lambda {\left(x\right)}^{4}$; the field equation is conditional on its absence (Section 6)
EFT truncation	postulate	two-derivative Fisher action, cutoff $L_{\mathrm{onset}}^{-1}$ (Section 6 and Section 9)
Lovelock’s hypotheses	postulate	uniqueness of the Einstein form (Section 6)
Clausius normalization	postulate	fixes $\kappa =8\pi G/c^{4}$ from $S_{\mathrm{BH}}$ (Section 6)
$a=\ell_{P}$	identification	patch spacing equals the Planck length (Section 7 and Section 9)
*G*	empirical input	sets physical units through $\ell_{P}$; leaves as it entered (Section 6)
$S_{\mathrm{BH}}=A/4\ell_{P}^{2}$	empirical input	supplies $c_{\mathrm{total}}$; calibrates $\lambda_{G}$ (Section 7)
Gaussian or binary patches	modeling choice	two statistics, two scales (Section 7 and Section 8)
$\lambda_{\mathrm{bin}}=\sqrt{2}\;a$	modeling choice	the largest scale binary patches can realize (derived bound); sitting at the edge is the choice (Section 8.3)
Cylinder-to-plane step	unproved step, tested	carries the cylinder bound 0.6389 to the plane; tested out of sample within a stated class of convergence forms; the open-strip bound 0.6135 does not use it (Section 8.6)
Minimum action = one distinguishable unit; evaluation scale Ωσ=1	postulate; modeling choice	give $\hbar_{q}=0.752$; unused downstream (Appendix F)
Estimate-level choices	modeling choice	uniform average over the phase offset (Appendix F); L=5 (Section 8.4); flat-patch reading of curved screens (Section 9.2)

**Table 2:** What follows from Table 1, and in what sense.

Result	Status	Depends on
Exponential density, Lorentzian kernel	derived	rows 1–4
Screen, metric, dimension rankB	measured	separation array, realizability
Lorentzian signature, v=1	follows	invariant-speed postulate
Form of the Einstein equation	derived	locality, no induced potential, EFT truncation, Lovelock, dimension 3+1
$\kappa =8\pi G/c^{4}$	normalized	Clausius; *G* is an input
$\lambda_{G}≃1.302\;\ell_{P}$	calibration	fitted to $S_{\mathrm{BH}}$
$c_{\mathrm{Ising}}^{\mathrm{per}}\left(5,\sqrt{2}\right)=0.660$	computed	$\lambda_{\mathrm{bin}}$ fixed in advance; L=5 chosen
$c_{\infty }≃1-1/\left(4\ln2\right)$	computed; exactness open	≥0.6135 rigorous; ≥0.6389 conditional on the cylinder-to-plane step; 0.63906±0.00008 by pre-registered extrapolation
Exponential noise spectrum	testable consequence	fixed-mean constraint (Appendix F)
B⪰0 on horizon data	falsifiable claim	realizability
$\hbar_{q}=0.752$	value at a chosen scale	minimum action, Ωσ=1

**Table 3:** Difficulties met by related programs and the corresponding step here.

Program	Difficulty	Here
AdS/CFT	needs AdS asymptotics and a boundary dual	area law used as a calibration only; no boundary assumed
RT, Van Raamsdonk, tensor networks	presuppose a bulk dual; network geometry chosen	geometry reconstructed from correlations, with a test that can fail
Cao–Carroll–Michalakis	distance map chosen; factorization assumed; spatial Einstein equation	kernel and distance map derived; no tensor factorization assumed, question deferred; spacetime form from Fisher geometry and Lovelock
Jacobson	presupposes horizons, Unruh temperature, and area law	form without horizons; Clausius fixes only κ
Verlinde	thermal force decoheres; screens postulated	no force; screen reconstructed
Causal sets	non-manifold-like orders dominate; dynamics incomplete	exact embeddability criterion; dimension as a rank

**Table 4:** The Gaussian residual at the candidate scales.

$\lambda /\ell_{P}$	Origin	$c_{G}$	$c_{\mathrm{total}}-c_{G}$
1.000	stipulated	0.3627	+0.2767
1.025	pairwise root (integral)	0.3832	+0.2561
1.3021	Gaussian root	0.6393	0.0000
1.4142	binary lattice choice	0.7543	−0.1150

**Figure A1:**
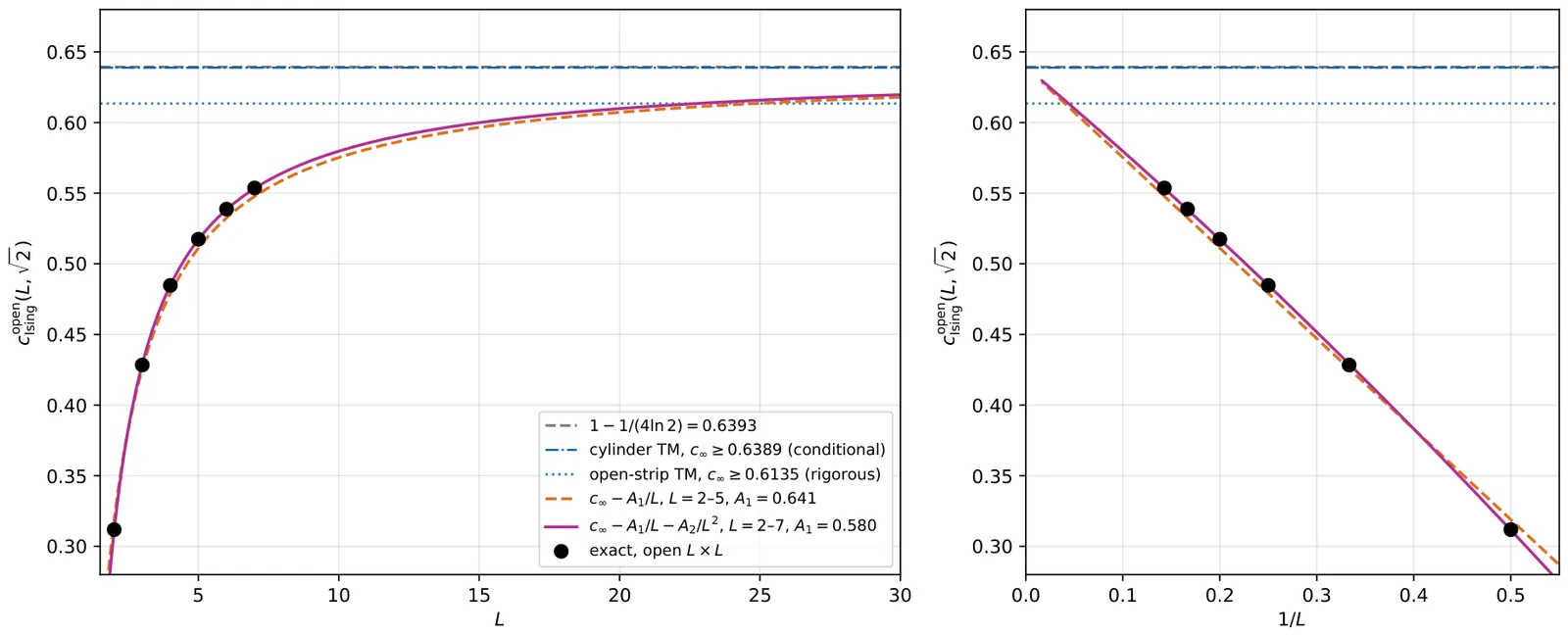
Finite-size behavior of $c_{\mathrm{Ising}}\left(L,\sqrt{2}\right)$ (open boundaries). Every symbol is a lower bound on $c_{\infty }$: exact enumeration (circles, L=2–7), the open-strip transfer-matrix bound (dotted, rigorous), and the cylinder bound (dash-dotted, conditional). Fits with $c_{\infty }$ fixed: $c_{\infty }-A_{1}/L$ over L=2–5 (dashed, $A_{1}=0.641$) and $c_{\infty }-A_{1}/L-A_{2}/L^{2}$ over L=2–7 (solid, $A_{1}=0.580$, $A_{2}=0.150$). Right: the same against 1/L.

## Data Availability

All code and data needed to reproduce the results are openly available at Zenodo, DOI: 10.5281/zenodo.23264575 (https://doi.org/10.5281/zenodo.23264575; all versions: 10.5281/zenodo.21857287), released under CC BY 4.0 with the code under the MIT License; no proprietary data were used. The archive’s README documents which script produces which figure, table, and numerical value, and the distinction between the open and periodic lattice conventions. For the two pre-registered tests of Appendix E it contains the frozen protocols with their SHA-256 hashes, the dated amendments, the frozen code, the production results with the hash of each results archive, and the recomputed verdicts. The archive also retains all 611 Wang–Landau runs of the previous version with their fit residuals (411 distinct parameter sets after de-duplication, with the acceptance criterion and the failing runs); no number in this version depends on them.

## Appendix A. Notation

**Table A1:** Symbols. Quantities measured in comparison units are in units of 1/Ω (Section 5).

Symbol	Meaning (Section)
P	Possibility space; its elements are positive-measure patches (Section 2).
$\omega_{i},\;\Omega \;\;\;\;\;\;\;\;\;\;\;\;\;\;\;\;\;\;\;$	Phase rate of patch *i* and its mean; $p\left(\omega \right)=\Omega^{-1}e^{-\omega /\Omega }$ (Section 3).
$\sigma ,\;\sigma_{ij}$	Comparison parameter and pairwise comparison interval, in comparison units (Section 2 and Section 4).
$\rho \left(\sigma \right),\;\rho_{ij}$	Two-point kernel $1/(1+\Omega^{2}\sigma^{2})$ and its matrix of values (Section 4).
ψ(σ)	Characteristic function ${\left(1-i\Omega \sigma \right)}^{-1}$; $\rho ={\left|\psi \right|}^{2}$.
$\lambda_{\sigma },\;\lambda ,\;\lambda_{0}$	Correlation scale $\Omega^{-1}$ (comparison units); length c/Ω; constant background length.
$\lambda_{G},\;\lambda_{\mathrm{bin}}$	Gaussian matching scale (1.302a) and binary lattice scale ($\sqrt{2}\;a$).
$B,\;P_{c}$	Centered Gram matrix $-\frac{1}{2}P_{c}ZP_{c}$ and centering projector; rankB is the embedding dimension (Section 5.1).
v,c	Invariant speed in comparison units (v=1) and in length units (Section 5.3).
$d_{ij}$	Length $c\;\sigma_{ij}$ (Section 5.3).
τ	Time coordinate: metric from the invariant-speed postulate, orientation from the entropy ordering (Section 10).
φ(x)	Fisher coordinate logΩ(x) (Section 6).
$D_{\mathrm{KL}},\;J_{ij}$	Kullback–Leibler divergence ∫pln(p/q), and its symmetrization 2(coshΔφ−1) (Section 6).
$f^{2},\;\kappa_{F}$	Decay constant $\hbar c/\lambda_{0}^{2}$; coupling $8\pi {\left(\ell_{P}/\lambda_{0}\right)}^{2}$ (Section 6.4).
$T_{\mu \nu }^{F},\;T_{\mu \nu }^{\left(\varphi \right)}$	Abstract and physical Fisher stress tensors.
$\rho_{F},\;p_{F},\;Q$	Fisher energy density, isotropic pressure, and ${\left|\nabla \varphi \right|}^{2}$.
$\chi ,\;\chi_{0},\;\chi_{★}$	Correlation field $\rho (\sigma_{*};\lambda \left(x\right))$, its constant value, and the kernel profile as the pairwise background (Section 6 and Appendix C.3).
a,x,L,N	Patch spacing ($=\ell_{P}$), λ/a, lattice side, $N=L^{2}$.
$L_{\mathrm{onset}}$	Scale below which no metric reconstruction exists (Section 9).
$s_{i},\;K_{ij},\;J_{ij}$	Ising variable, target correlation $\rho (d_{ij})$, and Ising coupling (Section 8).
$I_{\mathrm{pair}},\;I_{B},\;I_{G}$	Pairwise mutual information: Gaussian formula, binary (nats), and Gaussian (bits).
$c_{\mathrm{total}}$	Bekenstein–Hawking deficit 1−1/(4ln2).
$c_{2},\;c_{G},\;c_{\mathrm{Ising}},\;c_{\infty }$	Pairwise, Gaussian, and binary multi-information per patch; L→∞ limit of $c_{\mathrm{Ising}}$.
$c^{\mathrm{per}},\;c^{\mathrm{open}}$	Periodic (minimum-image) and open (straight) lattice conventions (Section 8).
$g\left(\theta_{0}\right),\;\hbar_{q},\;\tilde{E}$	Per-offset phase Fisher information, $\sqrt{\langle g\rangle }=0.752$, and energy in comparison units (Appendix F).
$\kappa_{H}$	Surface gravity.

## Appendix B. Numerical and Reproducibility Notes

Pairwise estimate.

The continuum form of the pairwise deficit per patch is

(A1) $$c_{2}\left(x\right)=\pi \int_{1}^{\infty }t\left[-\frac{1}{2}\log_{2}\left(1-\frac{x^{4}}{{\left(t^{2}+x^{2}\right)}^{2}}\right)\right]dt,$$

with root x=1.024712; the direct lattice sum $\frac{1}{2}\sum_{j\neq i}I_{\mathrm{pair}}\left(K_{ij}\right)$ on $Z^{2}$ has root x=0.898 (Appendix D).

Gaussian estimate.

$c_{G}=-\frac{1}{2N}\log_{2}\det K$ is computed on periodic L×L tori: $c_{G}\left(1\right)=0.36264691$, 0.36265595, and 0.36265627 at L=32, 128, and 2048, and L=128 and L=256 agree to six decimal places. The root is x=1.302051, stable from L=128. At x=2 one DFT eigenvalue is negative at L=8 and L=16; at the scales used here the smallest eigenvalues on L=256 are +0.172, +0.067, and +0.046 at x=1, 1.302, and $\sqrt{2}$.

Sphere spectra.

On a unit sphere with geodesic separations ($\lambda =\frac{1}{2}$), minspecB=−3.54, −6.79, and −13.39 at N=30, 60, and 120, and −444 at N=4000; minspecρ stays positive until N≈500 and reaches $-1.4\times 10^{-3}$ at N=4000 (check_sphere_largeN.py).

Scripts.

The robustness ratio (39) is computed by verify_matching_robustness_v2.py, the non-coordinate arrays of Section 5.1 by test_dimension_is_an_output.py, and the phase Fisher information of Appendix F by hbar_q_theta0.py.

## Appendix C. Correlation Field: Variational Structure

### Appendix C.1. Action Principle and Equation of Motion

From $d\rho /d\sigma =-2\Omega^{2}\sigma \rho^{2}$ and $\Omega^{2}\sigma^{2}=1/\rho -1$ the kernel satisfies

(A2) $$\frac{d^{2}\rho }{d\sigma^{2}}=2\Omega^{2}\rho^{2}\left(3-4\rho \right),$$

the equation of motion of

(A3) $$L\;\left(\rho ,\frac{d\rho }{d\sigma }\right)=\frac{1}{2}\;{\left(\frac{d\rho }{d\sigma }\right)}^{\;2}-V\left(\rho \right),\;V\left(\rho \right)=2\Omega^{2}\rho^{3}\left(\rho -1\right),$$

with V(0)=V(1)=0, V≤0 on [0,1], and minimum at ρ=3/4. The Hamiltonian $H=\frac{1}{2}{\left(d\rho /d\sigma \right)}^{2}+V$ vanishes at ρ(0)=1, $\rho^{\prime }\left(0\right)=0$, and on the orbit $\frac{1}{2}{\left(d\rho /d\sigma \right)}^{2}=2\Omega^{2}\rho^{3}\left(1-\rho \right)=-V$ identically: *V* was constructed from the kernel, so H=0 is an identity and not a selection principle. Given *V*, the uniqueness of the initial-value problem picks out the Lorentzian. Treating σ as an evolution parameter is a formal device, as in finding a soliton profile from a particle in an inverted potential; what it delivers is *V*, whose independent content is the fluctuation operator.

### Appendix C.2. The Equation of Motion in Length Units

With d=cσ and λ=c/Ω (Section 5.3),

(A4) $$\frac{d^{2}\rho }{dd^{2}}=\frac{2}{\lambda^{2}}\rho^{2}\left(3-4\rho \right),\;\rho \left(0\right)=1,\;\rho^{\prime }\left(0\right)=0,$$

whose zero-energy solution is $\lambda^{2}/\left(d^{2}+\lambda^{2}\right)$, with inflection at ρ=3/4, $d=\lambda /\sqrt{3}$.

### Appendix C.3. 1+1-Dimensional Field Extension

The kernel is a fixed two-point function. To let departures from it propagate, place a field χ(σ,τ) on the plane of pair separation σ and the time coordinate τ, with density

(A5) $$L\left[\chi \right]=\frac{1}{2v^{2}}\;{\left(\frac{\partial \chi }{\partial \tau }\right)}^{\;2}-\frac{1}{2}\;{\left(\frac{\partial \chi }{\partial \sigma }\right)}^{\;2}-V\left(\chi \right),$$

where *V* is the potential of Appendix C.1, and field equation

(A6) $$\frac{1}{v^{2}}\frac{\partial^{2}\chi }{\partial \tau^{2}}-\frac{\partial^{2}\chi }{\partial \sigma^{2}}+2\Omega^{2}\chi^{2}\left(4\chi -3\right)=0.$$

The relative minus sign in (A5) is the invariant-speed postulate, and since σ and τ carry the same unit, v=1 (Section 5.3). The static equation $\partial_{\sigma }^{2}\chi =V^{\prime }\left(\chi \right)$ has the opposite sign from (A2), because σ is the evolution parameter there and a spatial coordinate here. The kernel profile $\chi_{★}=1/\left(1+\Omega^{2}\sigma^{2}\right)$ therefore has zero energy density, $H\left[\chi_{★}\right]=\frac{1}{2}{\left(\partial_{\sigma }\chi_{★}\right)}^{2}+V\left(\chi_{★}\right)=0$, without solving the static equation; the spatial stress ${\left(\partial_{\sigma }\chi_{★}\right)}^{2}$ does not vanish.

Replacing $\partial_{\sigma }^{2}$ by $\nabla^{2}$ extends the pairwise equation to *D* dimensions by analogy (Section 11, limitation (iv)); in length units,

(A7) $$□\chi +\frac{2}{\lambda^{2}}\chi^{2}\left(4\chi -3\right)=0,\;□=c^{-2}\partial_{\tau }^{2}-\nabla^{2}.$$

$\chi_{★}$ solves (A7) when *d* is read as the time-like coordinate and fails for every *D* when *d* is radial; it is regular at the origin, so it is not a Green’s function and has no source. Fluctuations about it obey

(A8) $$□\epsilon +m_{\mathrm{eff}}^{2}\left(d\right)\;\epsilon =0,\;m_{\mathrm{eff}}^{2}\left(d\right)=V^{\prime \prime }\left(\chi_{★}\right)=\frac{12}{\lambda^{2}}\chi_{★}\left(d\right)\;\left[2\chi_{★}\left(d\right)-1\right].$$

The effective mass is set by the background at separation *d*, and it is independent of the wavelength of the perturbation: positive for d<λ, negative for d>λ, and $12/\lambda^{2}$ at the origin. Because $\chi_{★}$ is not a stationary point, the expansion keeps a source term, so $m_{\mathrm{eff}}^{2}$ is a local mass only in the sense of this operator. It is also no mass for the Fisher coordinate φ: *V* depends on φ explicitly through $\Omega^{2}=e^{2\varphi }$, and at $\chi_{★}=\frac{1}{2}$, $V^{\prime \prime }=0$ exactly, while the second derivative in φ at fixed σ is $\frac{1}{2}\Omega^{2}$. The Noether tensor of (A5) with v=1 gives energy density $\frac{1}{2}{\left(\partial_{\tau }\chi \right)}^{2}+\frac{1}{2}{\left(\partial_{\sigma }\chi \right)}^{2}+V$, conserved by (A6). The dynamics of Section 6 does not use this field theory; its source is built from $\partial_{\mu }\varphi$.

### Appendix C.4. The Two-Rate Kernel

With $\omega_{i}∼\mathrm{Exp}\left(\Omega_{i}\right)$ independently,

(A9) ρij=Ecos(ωi−ωj)σ=Re1(1−iΩiσ)(1+iΩjσ).

With $\Omega_{i,j}=\Omega_{g}e^{\pm \Delta \varphi /2}$ about the geometric mean and $w\equiv \Omega_{i}\Omega_{j}\sigma^{2}$,

(A10) $$\rho_{ij}=\frac{1+w}{{\left(1+w\right)}^{2}+4w\sinh^{2}\;\left(\Delta \varphi /2\right)}=\frac{1}{1+w}\;-\;\frac{w}{{\left(1+w\right)}^{3}}\;{\left(\Delta \varphi \right)}^{2}\;+\;O\left({\left(\Delta \varphi \right)}^{4}\right),$$

exact, even in Δφ, and equal to the Lorentzian at Δφ=0. The expansion is not an action for φ, for two reasons. At fixed σ, $w=e^{2\bar{\varphi }}\sigma^{2}$, so the coefficient depends on $\bar{\varphi }$ and breaks the shift symmetry: σ acts as a spurion (a fixed parameter that breaks the symmetry). And it describes a pair at a chosen separation; converting it into a one-point density is the locality assumption.

## Appendix D. Pairwise Mutual-Information Approximation

$I_{\mathrm{pair}}\left(\rho \right)=-\frac{1}{2}\log_{2}\left(1-\rho^{2}\right)$ is exact for two jointly Gaussian variables and depends on the correlation alone. With patch density $a^{-2}$, excluding the self-term by the lower limit r=a and treating the screen as locally flat,

(A11) $$c_{2}\left(\lambda \right)=\frac{1}{2}\int_{a}^{\infty }\frac{1}{a^{2}}I_{\mathrm{pair}}\left(r\right)\;2\pi r\;dr,$$

which is the integral of Appendix B in t=r/a, x=λ/a. Setting $c_{2}\left(x\right)=c_{\mathrm{total}}$ gives x≃1.0247. The integral replaces a lattice sum, and the root depends on the cutoff: the direct sum over $Z^{2}$ gives x=0.898, and a cutoff at $a/\sqrt{\pi }$ gives 0.857. The pairwise sum counts information shared by three or more patches once per pair; here $c_{2}$ exceeds $c_{G}$ (at x=1, $c_{2}\approx 0.593$ against $c_{G}\approx 0.363$).

## Appendix E. The Thermodynamic Limit of c_Ising_: Methods and Controls

Marginality.

For an isotropic profile $\rho ∼d^{-\alpha }$ on a two-dimensional screen, the correlation accumulated by one patch to separation *L* is

(A12) $$\Sigma \left(L\right)\;=\;\sum_{0<d<L}\rho \left(d\right)\;∼\;\left\{\begin{array}{cc} L^{2-\alpha }, & \alpha <2,\\ \ln L, & \alpha =2,\\ \mathrm{const}, & \alpha >2, \end{array}\right.$$

so the kernel’s α=2 is the marginal case, reached with no tuning. The analogy with self-organized criticality [53] is loose, since that concerns driven dynamics. The statement concerns the correlations; the couplings fitted here are not shown to fall as $d^{-2}$.

Finite-size sequence.

With open boundaries every entry is a lower bound on $\max_{J}c_{\mathrm{Ising}}\left(L,\sqrt{2}\right)$: by convex duality $c_{\mathrm{Ising}}=\max_{J}\left[1-\left(\log Z\left(J\right)-J\cdot K\right)/\left(N\ln2\right)\right]$, so every trial *J* gives a lower bound. The partition function is enumerated exactly on the hard-constraint support of Section 8.3: 2030, 60,232, 3,858,082, and $4.47\times 10^{8}$ configurations at L=4–7, against $2^{L^{2}}$. At the optimum the correlation residual is below $10^{-10}$, the primal and dual values agree to $10^{-9}$, and at L=2–5 the values agree with enumeration over all $2^{L^{2}}$ configurations:

(A13) $$c_{\mathrm{Ising}}^{\mathrm{open}}\left(L,\sqrt{2}\right):\;L=2:\;0.3119,\;L=3:\;0.4284,\;L=4:\;0.4848,\;L=5:\;0.5174,\;L=6:\;0.5387,\;L=7:\;0.5537.$$

Each entry also bounds the limit. Tiling a kL×kL open box with L×L tiles and dropping the constraints between tiles can only raise the maximum entropy, so $H_{\max}\left(kL\times kL\right)\leq k^{2}H_{\max}\left(L\times L\right)$, and $c_{\mathrm{Ising}}^{\mathrm{open}}\left(L,\sqrt{2}\right)\leq c_{\infty }$ for every *L*. Sampling-based estimates of logZ at L≥6 fall below the exact value at L=6 and are not used.

Fits.

With $c_{\infty }=1-1/\left(4\ln2\right)$ fixed, the one-parameter form $c\left(L\right)=c_{\infty }-A_{1}/L$ over L=2–5 gives $A_{1}=0.641$, and the two-parameter form $c_{\infty }-A_{1}/L-A_{2}/L^{2}$ over L=2–7 gives

(A14) $$A_{1}=0.580,\;A_{2}=0.150$$

(Figure A1). The per-point coefficient $(c_{\infty }-c\left(L\right))L$ is 0.655, 0.633, 0.618, 0.609, 0.604, and 0.599 at L=2–7 and falls smoothly, so the exact sequence places $A_{1}$ near 0.58, well below ln2=0.693.

*An out-of-sample check.* Before L=7 was computed, three extrapolation forms fitted on L=3–6, each corrected by its own error on the exact Gaussian sequence, predicted c(7)=0.5536993–0.5537044 with tolerances of $2\times 10^{-5}$; the exact value is 0.5537034. Refitting with L=7 included and removing each form’s Gaussian-control error gives $c_{\infty }=0.63902\pm 0.00029$, the uncertainty dominated by how well the Gaussian correction transfers to the binary case. This check was fixed in advance with the test below; it is consistent with both 1−1/(4ln2) and Equation (42) and cannot separate them.

Transfer-matrix bounds.

The transfer matrix of a strip of width *M* gives its entropy per site exactly; a Newton step finds the couplings that maximize the entropy per site $h_{S}$ subject to the Lorentzian correlations on a set *S* of separations, and $c_{\infty }\geq 1-h_{S}/\ln2$, since each added constraint can only lower the maximum entropy. Rows are restricted to the hard-constraint support of Section 8.3, which every admissible law satisfies, so the restriction leaves the bounds valid; on that support the axial correlations at separation 2a are implied by those at *a*. The code reproduces brute-force partition functions on small tori to $10^{-15}$, its gradients agree with finite differences to $10^{-10}$, and on all $2^{M}$ rows it reproduces the six-shell value 0.60465 at M=6.

*Open strips.* The entropy per site of any translation-invariant law on the plane is at most the maximal row entropy of an open strip, per site, under the strip-averaged constraints, and by weak duality that is at most the dual value at any coupling. With M=12 and every separation with 0≤Δy≤3, |Δx|≤5 constrained, the dual value gives Equation (40), $c_{\infty }\geq 0.6135$, with no limit assumption. The edge columns of an open strip cost entropy, so this bound tightens as O(1/M).

*Cylinders.* On a periodic cylinder of circumference M≥2D+1, constraining every separation with $0\leq \Delta y\leq R_{y}$ and |Δx|≤D individually, $R_{y}=D=4$ at M=10 gives c≥0.63886, Equation (41); the larger set $R_{y}=5$, D=4 at M=11 gives 0.63894. The cylinder is not a subsystem of the plane, since it carries wrapped correlations, so the step to the plane is assumed: at fixed constraint set the cylinder value converges to the plane value. Three checks support it. For the sets with D=4 computed at several widths, the values at M=10–13 agree to $\pm 3\times 10^{-5}$. Transposing the constraint set, so that the long separations run along the infinite direction, changes the value by $2\times 10^{-5}$. Both the binary values and the exact Gaussian maximum-entropy law on the same cylinders read high by about $7\times 10^{-4}$ at M=2D+1, which is why that width is not used; at larger widths both vary by about $5\times 10^{-5}$, though their patterns differ in detail (from M=10 to 11 the binary values rise by $10^{-5}$ while the Gaussian ones fall by $10^{-4}$). At small *M* the step fails visibly: M=5 gives 0.650, above 1−1/(4ln2).

*The unconstrained tail: a pre-registered test.* Separations outside the set of Equation (41) are not constrained. For the Gaussian maximum-entropy law, constraining them raises *c* by $2.0\times 10^{-4}$. Before the larger sets were computed, the following were fixed and archived: the statistic, the binary-to-Gaussian ratio ρ of the increments from S(4,4) to S(5,5), each measured along the infinite direction at fixed width (M=10 and 12); a band, 0.66≤ρ≤1.19, inside which the extrapolation counts as confirmed; four trend models for the ratio beyond S(5,5); and the decision rules. The measured ratio is ρ=0.864 (0.833 against width-matched Gaussian increments), the plane value at S(5,5) is 0.63900, and the trend models give $c_{\infty }=0.63904\pm 0.00005$, with an uncertainty dominated by the width dependence. If $c_{\infty }$ equaled 1−1/(4ln2), the binary ratio beyond S(5,5) would have to be 8.1.

*The cylinder-to-plane step: a pre-registered test.* The width uncertainty above was taken from circumferences 10–13. A second test, also fixed and archived before any new value was computed, extended the binary cylinders: S(4,4) to M=15, S(5,4) to M=12, and the cheaper sets S(2,4), S(3,4), and S(2,3) to M=17. Convergence was modeled with inverse powers of *M*, a free power law, an exponential with a parity oscillation, and separate fits to even and odd *M*; a model counted only if it predicted its own largest-*M* point. Every new value fell inside the band predicted in advance from M≤12, and none drifted outside every model. The plane values lie within $6.5\times 10^{-5}$ of the cylinder values at M=10–12, and the correction to the S(5,4) value used above is $+2.5\times 10^{-5}\pm 7.7\times 10^{-5}$; including it gives Equation (42), $c_{\infty }=0.63906\pm 0.00008$. If $c_{\infty }$ equaled 1−1/(4ln2), the binary ratio beyond S(5,5) would have to be 7.4. The binary values at D=4 still rise by $∼10^{-5}$ per unit of *M* at the largest widths, which the models absorb; a correction converging as slowly as $M^{-1/2}$ would escape this test, which therefore establishes the step only within the stated class of forms.

*Conventions.* The bound is periodic and the sequence open. Comparing their limits is legitimate: $I_{B}∼K^{2}∼d^{-4}$ is summable in two dimensions, so boundaries contribute $O(L^{-1})$ per site. The exact periodic values are 0.6601, 0.6536, and 0.6365 at L=5–7; they are not monotone toward the limit at these sizes and are not used for it.

Computational limits.

On the support, exact enumeration reaches the open 7×7 lattice ($4.47\times 10^{8}$ admissible configurations, about six CPU-hours) and the 7×7 torus. The largest cylinder set used, $R_{y}=D=5$ at M=12, needs $1.6\times 10^{8}$ transfer-matrix states after reduction by the lattice symmetries.

## Appendix F. Noise and the Quantum of Action

Standing waves.

Two highly correlated patches at abstract separation *ℓ* can be modeled by fixed ends, ϵ(0,τ)=ϵ(ℓ,τ)=0, so the fluctuation modes of Appendix C.3 are $\epsilon_{n}=A_{n}\sin\left(n\pi \sigma /\ell \right)\cos\left(\nu_{n}\tau \right)$ with $\nu_{n}=n\pi /\ell$: discrete frequencies from boundary conditions alone, the analog of de Broglie’s condition [54].

Noise spectrum.

The difference $\omega_{i}-\omega_{j}$ of two independent exponentials is Laplace-distributed, so the noise spectrum of the coherence (43) is the density of |Δω|, $S\left(\omega \right)=\Omega^{-1}e^{-\omega /\Omega }$, the Wiener–Khinchin transform of the kernel. With the time–bandwidth product [55] the minimum detectable squared amplitude scales as S(ω)ω, so the detection threshold is ${\tilde{E}}_{\mathrm{noise}}\propto \omega^{3}e^{-\omega /\Omega }$, with $\tilde{E}$ an energy in comparison units. It is not linear in ω, so it gives no quantum of action. The exponential tail is itself a consequence of the fixed-mean constraint, so the spectrum is a testable consequence of the inference principle: a fixed variance would give a Gaussian tail, and a Lorentzian spectrum, easily confused with it because the kernel is Lorentzian in σ, differs from the exponential by a factor of 14 at ω=7Ω. Direct sampling of |Δω| reproduces the exponential to 0.004 in density.

Minimum action from Fisher information.

Let $\theta_{0}$ be an unknown common phase offset of a pair; how well a coherence measurement fixes it sets the smallest resolvable phase interval. (This Fisher information is distinct from the Fisher metric of the rate parameter, $g_{\varphi \varphi }=1$, of Section 6). Three steps:

(i) For a mode of frequency ν the phase advances as $\theta_{0}=\nu \tau$, so by reparametrization covariance $g_{\tau \tau }=\nu^{2}g_{\theta_{0}\theta_{0}}$. Reading the pair information as a per-mode quantity is a second use of the locality assumption.
(ii) The Cramér–Rao bound [56] makes the smallest resolvable interval $\Delta \tau_{\min}=1/\sqrt{g_{\tau \tau }}$.
(iii) The postulate: one distinguishable unit of action per resolvable interval, ${\tilde{E}}_{\min}\Delta \tau_{\min}=1$, a counting statement in the sense of statistical distance [57,58].

Together,

(A15) $${\tilde{E}}_{\min}\left(\nu \right)=\hbar_{q}\;\nu ,\;\hbar_{q}=\sqrt{g_{\theta_{0}\theta_{0}}},$$

so the minimum energy is linear in the frequency. In physical units the action unit is *ℏ*; $\hbar_{q}$ is the minimum action in comparison units, and only the linearity is used in Section 6.4.

Evaluating the constant.

The observable is $X=\cos(\theta +\theta_{0})$ with θ=Δωσ. The wrapped density of θ has Fourier coefficients E[cosnθ]=ρ(nσ), so with X=cosϑ the density of the observable is exactly

(A16) $$P\left(\vartheta \;|\;\theta_{0}\right)=\frac{1}{\pi }\left[1+2\sum_{n\geq 1}\rho \left(n\sigma \right)\cos\left(n\vartheta \right)\cos\left(n\theta_{0}\right)\right],\;\vartheta \in \left[0,\pi \right],$$

equivalently a wrapped Laplace density folded under ϑ→−ϑ. The per-offset information $g\left(\theta_{0}\right)=\int_{0}^{\pi }{\left(\partial_{\theta_{0}}P\right)}^{2}/P\;d\vartheta$ depends on $\theta_{0}$: at Ωσ=1 it is 0, 0.644, and 0.841 at $\theta_{0}=0$, π/4, and π/2. It vanishes at $\theta_{0}=0$ because *X* is even in the offset there. The offset is unknown, and under the maximum-entropy ensemble it is uniform on [0,2π). The uniform law carries no Fisher information of its own, so by the Bayesian Cramér–Rao (van Trees) inequality [59] the mean-square error of the offset is bounded by 1/〈g〉; the average is therefore taken over *g*,

(A17) $$g_{\theta_{0}\theta_{0}}={\langle g\left(\theta_{0}\right)\rangle }_{\theta_{0}}=0.566,\;\hbar_{q}=\sqrt{g_{\theta_{0}\theta_{0}}}=0.752\;\left(\Omega \sigma =1\right),$$

a pure number. (Averaging $\sqrt{g}$ instead gives 0.72). $g_{\theta_{0}\theta_{0}}$ lies below the unwrapped bound ${\left(\Omega \sigma \right)}^{-2}=1$. The evaluation point is a choice (Table 1): $\sqrt{g_{\theta_{0}\theta_{0}}}$ is 1.77, 0.752, and 0.263 at $\Omega \sigma =\frac{1}{2}$, 1, and 2, and the counting postulate contains no σ to fix it. Ωσ=1 is the one scale the kernel distinguishes.
